# AAA Revisited: A Comprehensive Review of Risk Factors, Management, and Hallmarks of Pathogenesis

**DOI:** 10.3390/biomedicines10010094

**Published:** 2022-01-02

**Authors:** Veronika Kessler, Johannes Klopf, Wolf Eilenberg, Christoph Neumayer, Christine Brostjan

**Affiliations:** Department of General Surgery, Division of Vascular Surgery, Medical University of Vienna, Vienna General Hospital, 1090 Vienna, Austria; veronika.kessler@gmail.com (V.K.); johannes.klopf@meduniwien.ac.at (J.K.); wolf.eilenberg@meduniwien.ac.at (W.E.); christoph.neumayer@meduniwien.ac.at (C.N.)

**Keywords:** abdominal aortic aneurysm, risk factors, pathogenesis, review, treatment

## Abstract

Despite declining incidence and mortality rates in many countries, the abdominal aortic aneurysm (AAA) continues to represent a life-threatening cardiovascular condition with an overall prevalence of about 2–3% in the industrialized world. While the risk of AAA development is considerably higher for men of advanced age with a history of smoking, screening programs serve to detect the often asymptomatic condition and prevent aortic rupture with an associated death rate of up to 80%. This review summarizes the current knowledge on identified risk factors, the multifactorial process of pathogenesis, as well as the latest advances in medical treatment and surgical repair to provide a perspective for AAA management.

## 1. Introduction

An aneurysm is a persistent and localized weakening and dilation of a blood vessel, typically an artery [1]. An abdominal aortic aneurysm (AAA) is, therefore, an irreversible dilation of the abdominal aorta between the diaphragm and the iliac bifurcation [2]. AAAs are typically ‘true aneurysms’, characterized by involvement and dilation of all three layers of the vascular wall. In contrast, a pseudoaneurysm or ‘false aneurysm’ caused by an arterial injury, such as a puncture or dissection, is hallmarked by blood infiltrating between the wall layers [2].

AAAs that are located below the renal arteries, i.e., infrarenally, account for about 80% of cases. The remainder are juxtrarenal, pararenal, and suprarenal AAAs that involve the renal arteries or occur above them, respectively. Typically, the wall dilation in AAAs involves the whole circumference of the aorta (‘fusiform’). Other morphological types such as saccular AAAs, where only part of the circumference is involved, are less common [3,4]. 

Clinically, no uniform definition of an AAA is universally accepted. The most commonly used definition is a maximum infrarenal abdominal aortic diameter of ≥30 mm, measured by ultrasonography or computed tomography angiography (CTA) [4,5]. This threshold is based on measurements of healthy infrarenal aortic diameters and is usually more than two standard deviations above the mean diameter of 17.9–19.3 mm for men [6,7]. However, this cutoff might not be appropriate for patients whose infrarenal aortic diameters differ from these dimensions, for instance, due to their height [5]. An alternative definition relates the maximum infrarenal aortic diameter to its expected normal value or to the diameter of the adjacent (i.e., the undilated suprarenal) aorta. Accordingly, a dilation should be considered aneurysmal when this ratio exceeds 1.5 [1,8]. This definition might be more useful for women, whose infrarenal aortic diameters measure a mean of 15.5–16.7 mm, as well as for distinct ethnic populations with deviating diameter values, and it can also be applied to other aneurysms [9,10].

## 2. Epidemiology

Lifetime risk of AAA is approximately 1 in 17 in the general population and up to 1 in 9 for current smokers [11]. A 2013 meta-analysis analyzing 56 studies of the years 1991–2013 found prevalence rates of 6.0% for men and 1.6% for women [12]. However, reported incidence and prevalence rates show a high degree of heterogeneity, owing to the specific definition of AAA used by the original studies and characteristics of the study populations, such as world region or population age [13]. Mortality of AAA patients is increased when compared to the general population, in both treated and untreated patient groups [14]. Rupture is the main and most often lethal complication of AAAs, but the most common causes of death for AAA patients are cardiovascular events [15,16]. Among AAA patients, women are more likely to die from an AAA-related death (i.e., rupture), while men die from cancer more often than from ruptures [17]. The mean time between a scan with no detected aortic abnormalities and AAA-related death is about 10 years (range 3.8–15.0) [18].

Analyses of historical data show a marked increase of AAA incidence, prevalence, and mortality during the 20th century. Over the past two decades, numbers have been on the decline in most countries, especially in Western Europe and North America [19,20]. Pooled prevalence rates have decreased from 5.7% in 1988–1998 to 2.8% in 2011–2013 [12]. AAA mortality has declined, as well, in most developed countries, with males and those under the age of 75 showing the greatest improvement [15,21]. The rise and fall of AAA-related mortality rates in developed countries correlate strongly with the changes in smoking prevalence [11,15]. Notably, AAA mortality in men is declining in many countries, particularly in the United States, the United Kingdom, and the Netherlands, while it seems to be on the rise among men and women in Hungary, Romania, and Japan [22].

## 3. Clinical Presentation and Course of Disease

AAAs are typically asymptomatic until they rupture. AAAs cause unspecific symptoms, if any, such as abdominal tenderness or pain radiating towards the back or to the genitals [23]. A pulsating abdominal mass may indicate the presence of an AAA, but abdominal palpation is inherently insensitive for detection of AAAs [24]. Symptoms may also be caused by complications such as compression of nearby organs or embolic events, but approximately half of patients have a ruptured aneurysm as their cause of primary presentation [25,26].

### 3.1. AAA Growth Rate

Disease progression is commonly non-linear with intermittent periods of aneurysm growth. The majority of AAAs enlarge over time and only a minority of patients show no detectable growth at all [27]. Mean growth rates range between 2.2 and 2.8 mm per year [28,29,30,31] and do not seem to have changed over the past 25 years [32]. Growth rates vary greatly between patients, however, and every second AAA never progresses to surgery or rupture [33]. A multicenter study found that in 0.9% of patients, the aorta progressed from a subaneurysmal aortic dilation (diameter 2.5–2.9 cm) to the threshold for elective surgical intervention of 5.5 cm in as little as five years, while another quarter of patients reached this size after 10–15 years [34].

Smoking is the most important modifiable risk factor influencing growth rates [28,30,31]. Current smoking increases growth rates by about 20%. Notably, large aneurysms are also known to grow faster: the baseline aneurysm diameter is strongly associated with growth. The mean estimated growth rate increases by 0.59 mm per year for every 0.5 cm increase in the baseline diameter. For instance, an aneurysm of 3.0 cm in diameter grows an average of 1.3 mm/year, while a 5.0 cm aneurysm grows 3.6 mm per year [35]. Other factors influencing growth rates will be further explored in the chapter on risk factors.

### 3.2. Aneurysm Rupture

Rupture is the main complication of AAAs and is associated with mortality rates of 65–80% overall [12,36]. Approximately 150,000–200,000 deaths per year worldwide can be attributed to AAA rupture [37,38]. Incidence and mortality rates of ruptured AAAs (rAAAs) have been declining over the past decades, while diagnoses of intact AAAs have increased [25,39]. These developments correspond with the decline in smoking rates among men, as well as the implementation of screening programs and subsequent uptake in elective repairs [15,20]. Less common complications of AAAs include aortoenteric or aortocaval fistulae and iliac vein compression resulting in deep vein thrombosis or emboli [40,41,42].

The clinical presentation of a ruptured AAA is usually dramatic with sudden-onset abdominal, chest, or back pain, and hypotension or hemorrhagic shock due to massive intra-abdominal bleeding [43]. However, the classic triad of abdominal or back pain, shock, and a palpable clinical mass is only present in about 50% of rAAA cases, causing frequent misdiagnosis as myocardial infarction, ureteric colic, or perforated gastrointestinal ulcer [44]. The rupture varies in location and extent, but if not surgically repaired immediately, it typically leads to fatal internal bleeding [26]. Retroperitoneal (posterolateral) rupture is observed most frequently, at about 80%, where bleeding may be temporarily restrained by a tamponade effect [40]. Around 20% of ruptures occur anteriorly, with mostly rapid intraperitoneal bleeding and patient death. Rupture in combination with the formation of an aortocaval fistula (3–4%) or a primary aortoduodenal fistula (<1%) are comparably rare events [40].

AAA diameter is the strongest predictor of rupture. The link between diameter and rupture is well established and provides the basis for surveillance intervals for patients with small AAAs [35]. A 2013 meta-analysis found that rupture rates doubled for every 0.5 cm increase in diameter [28]. Furthermore, rapid aneurysm growth of over 2 mm per year significantly predicts AAA-related clinical events [33]. Other factors associated with increased risk of rupture are increased age, female sex, current smoking, and untreated hypertension, as well as aneurysm morphology [30,45].

## 4. Risk Factors and Comorbidities

Besides age and male sex, the strongest predictors of AAA are smoking and atherosclerotic cardiovascular diseases. Notably, in contrast to its role in atherosclerotic disease, type II diabetes is associated negatively with AAA.

### 4.1. Influence of Age and Sex

Age is one of the most important risk factors for the development of an AAA. Compared to a 40–44-year-old man, the risk is increased almost 200-fold for a 75–79-year-old man (0.83 versus 164 per 100,000) [20]. Fewer data exist regarding AAA incidence in women, but meta-analyses of epidemiological data and population screenings indicate that the AAA risk increases with age for women in a similar fashion, albeit on a lower level [12,46]. AAA prevalence rates for women are approximated at around 0.74–1.6%. However, it is estimated that with an adapted threshold value of 26–27 mm for women, based on a 50% increase in size from the average baseline, the AAA prevalence in 70-year-old women would more than double [47,48].

As for men, increased age and current smoking are the strongest risk factors for women [49,50]. Women tend to develop AAAs later in life compared to men, but the disease progresses more aggressively [51]. Thus, AAAs in women grow faster and their rupture risk is about four times higher than in men [28,52,53]. Women’s AAAs tend to rupture at smaller diameters than men’s [30,54,55], and although prevalence is 4–6 times lower than in men, almost every third patient presenting to the hospital with a ruptured AAA is female [56].

### 4.2. Ethnicity and Socioeconomic Factors

AAA prevalence is higher among men of Caucasian descent than among African-American, Hispanic, and Asian men, and these differences persist even after adjusting for all other known risk factors [5,49,57,58]. While white men in the USA are more likely to develop an AAA, non-white ethnic groups exhibit worse outcomes [59,60,61,62]. A multitude of individual and environmental factors might help explain these differences, but the proportion of their respective impact is not without controversy [57,63]. It seems that, rather than anatomical or biological differences, disparities between different ethnicities’ courses of disease and outcomes may be mediated by the level of education, socioeconomic status, and disposable income [64,65]. These factors have been shown to be associated with screening attendance and AAA prevalence [66,67], as well as with rupture rates [59,61,68], even after adjusting for sex, age, and comorbidities. Educational level influences how patients view and manage their own health. For instance, lower education reduces adherence to preventive medication and decreases the success rate of smoking cessation [69,70]. A low educational level does not affect survival after AAA repair, but low disposable household income is associated with increased mortality after AAA repair, both short-term and over one year, regardless of ethnicity [68,71]. On a nonindividual level, discrepancies in management of risk factors and medical management by health care personnel may hold a certain influence [60].

### 4.3. Family History and Genetic Influences

Individuals with a first-degree relative with an AAA (i.e., parent, sibling, or offspring) have an increased risk of developing an AAA themselves (odds ratio, OR 1.96–3.8) [72,73,74]. Familial AAA cases tend to present at a younger age, and are associated with an increased growth rate and higher rupture rate compared to sporadic cases, even though aneurysm morphology does not seem to be different [75,76].

About 6–20% of AAA patients have a positive family history [73,77,78,79], but except for rare hereditary diseases, such as Marfan syndrome or Ehlers–Danlos syndrome [80], no distinct inheritance patterns have been identified to help explain familial AAA clusters. Two large twin studies have concluded that genetic factors may contribute as much as 77% to development of an AAA, with the remaining 23% attributed to individual and environmental factors, such as smoking [81,82]. In recent years, efforts have been made to identify genetic and epigenetic factors (including microRNAs and long noncoding RNAs) that contribute to AAA pathogenesis [83,84,85]. With respect to single nucleotide polymorphisms, gene variations associated with AAA were found to be manifold and partly shared with cardiovascular disease [84,85]. Thus, polygenic effects are likely to contribute to AAA pathogenesis. As of yet, the identified factors explain but a fraction of the heritability of aneurysmal disease, but they may help to elucidate pathophysiological processes and potentially serve as biomarkers or therapeutic targets in the future [86,87].

### 4.4. Smoking

Smoking is widely accepted as the key modifiable risk factor for AAA, as it contributes significantly to development, growth, and rupture of AAAs. It is estimated that 75% of all AAAs larger than 4.0 cm in diameter can be attributed to smoking [88]. In a USA screening study with 3.1 million participants, 80.2% of all AAAs were diagnosed in smokers when they comprised 42.8% of the cohort [49]. The effect of smoking on AAA development seems to be even stronger in women [48,89,90]. While a 10-fold, as opposed to 3-fold, risk to develop AAA is reported for current versus past female smokers, risk factor exposure in the pre- and/or post-menopausal period is rarely discriminated in studies on women with aneurysmal disease, and might offer further pathomechanistic insight [89].

Reported ORs for AAA development between smokers and nonsmokers range between 2.3 and 13.72 [57,77,91,92], making the association even stronger than between smoking and coronary heart disease or chronic obstructive pulmonary disease (COPD) [93,94]. COPD itself has also been suggested to be positively associated with AAA [95,96], independently of smoking [97]. While COPD does not seem to increase AAA growth [98], it does increase the risk of rupture at smaller diameters [99,100,101]. Smoking increases AAA growth rate by 15–24% and is associated with an increased risk of rupture, regardless of diameter (hazard ratio (HR) 2.02) [28,30,31,32].

The effect of nicotine consumption on AAA development seems to be dose-dependent, i.e., both duration and amount of smoking matter [49,65,92]. Every year of smoking increases the relative risk (RR) of AAA by 4% [102] and a recent meta-analysis found a summary RR of 1.87 per 10 cigarettes per day [103]. Current smokers have a particularly high risk, with an HR of 5.55 compared to an HR of 1.91 for former smokers [57]. Smoking cessation reduces the risk of AAA formation, aneurysm growth, and lowers rupture rates [30,104]. Reports indicate that it may take about ten years for the excess risk for former smokers to be halved, and that it does not approach the risk of never-smokers until at least 25 years after quitting [90,94,103].

### 4.5. Atherosclerosis and Cardiovascular Diseases

Atherosclerotic cardiovascular diseases (CVDs) and AAAs often coincide, and their intricate relationship entails commonalities, as well as distinct aspects in risk factors and pathomechanisms. The most striking discrepancy in risk profiles is the contrasting role of diabetes mellitus [105]. Generally, CVDs and AAAs are considered distinct disease entities [41,106].

AAA patients often have cardiovascular comorbidities and vice versa. High coprevalence rates of AAA and coronary artery disease (CAD) [107], peripheral artery disease (PAD) [11,108], and cerebrovascular disease [109] are well established [16,110]. Prevalence rates of AAA in CVD patients are higher than among the general population, with a greater effect of PAD than CAD or carotid atherosclerosis [49,110]. In addition to mere coprevalence, it has been shown that a history of atherosclerotic disease increases the risk of developing an AAA about fourfold, and this risk is amplified by having multiple or severe CVDs [77,107]. It is estimated that up to two thirds of AAA patients have a relevant CAD, and AAA prevalence is higher in patients with three-vessel disease versus a lower-degree CAD [111,112]. An Italian population-based study found the highest AAA prevalence in the subgroups with high cardiovascular risk, previous myocardial infarction, and stroke [113].

No consistent and robust association between CAD or PAD and increased or reduced AAA growth or rupture rates has been found [27,99,114,115]. However, AAA patients seem to be prone to higher disease severity of atherothrombotic diseases and are at risk for cardiovascular events [110]. Polyvascular disease, i.e., atherothrombosis in multiple vascular beds, was found to be twice as common in AAA patients as in non-AAA patients with other CVDs or with at least three cardiovascular risk factors (31.6% versus 15.5%). In the same study, AAA patients were found at greater risk for newly diagnosed or worsening PAD and showed increased rates of hospitalization for atherothrombotic events and revascularization procedures at the 1-year follow-up [110]. A patient with a small AAA is about 1.5 times more likely to suffer a cardiovascular event than a patient without an aortic aneurysm, and has a 3% risk of cardiovascular death per year [16,116]. Thus, despite high mortality rates of AAA ruptures, cardiovascular events remain among the most common causes of death in AAA patients [117,118].

### 4.6. Arterial Hypertension and Dyslipidemia

Pre-existing arterial hypertension increases the risk of developing an AAA significantly, with a stronger effect on women than on men [119]. While hypertension does not seem to be associated with AAA growth, hypertensive AAA patients do show an increased rate of aneurysm rupture [28,31,120]. Reports indicate a dose-dependent relationship between blood pressure and AAA, both for formation and rupture [30,119], and diastolic blood pressure might have a more pronounced effect than systolic [31]. It is estimated that the risk of rupture increases 1.11-fold for every 10 mmHg increase in mean blood pressure [30].

Dysregulated serum levels of total cholesterol, triglycerides, and lipoproteins are major risk factors for atherosclerosis and CVDs. Regarding AAA development, high serum high-density lipoprotein (HDL) levels have an undisputed protective effect (OR 0.7–0.83) [11,121,122], while data are not as homogenous with regards to total cholesterol (TC), low-density lipoprotein (LDL), or triglyceride levels. An analysis of historical and current laboratory data of patients from 12 years before their initial AAA diagnoses found that prior elevated TC, LDL, and triglyceride levels were significantly associated with current AAA (ORs 1.9, 2.3, and 1.9, respectively) [77]. Two meta-analyses found conflicting results, with one confirming that increased LDL levels are associated with AAA presence [121], while the other found no such association for LDL, but found a significant effect on AAA development for elevated total cholesterol levels [122]. Dyslipidemia does not seem to influence AAA growth or rupture rates [27].

Methodological differences between clinical studies and interindividual variations in lipid metabolism could contribute to contradictory results [41]. Some authors suggest that the traditional classification of cholesterol and triglycerides could be insufficient to determine the true contribution of dyslipidemia to AAA development [41,123].

### 4.7. Obesity and Lifestyle Habits

Studies investigating the influence of obesity and high body surface area on AAA development and growth have yielded conflicting results [124]. Both waist circumference and waist-to-hip ratio as measures for intra-abdominal fat mass, i.e., visceral adiposity, have been reported to be associated with the risk of AAA [125,126]. This association was stronger in AAAs with a diameter above 40 mm, and seemed to increase linearly with waist circumference up to a certain point [127,128]. However, no clear association of visceral adiposity on AAA development or growth has been found [129]. A high body mass index may, however, be associated with reduced AAA mortality [15,20]. This phenomenon known as the ‘obesity paradox’, i.e., an association between cardiovascular risk factors and an improvement in clinical outcomes, has been observed in other diseases as well [130]. Obstructive sleep apnea, often associated with adiposity, is common in AAA patients and may be a risk factor for aneurysm growth, but further studies are required to elucidate this relationship [131,132].

Regular exercise is generally safe for, and well tolerated by, patients with small AAAs and does not influence growth rates, but may reduce the risk of AAA development [133,134,135]. A well-balanced diet may reduce the risk of AAA, but studies analyzing diet quality in AAA patients are scarce [136,137]. A deficiency of vitamin D has been associated with AAA presence [138], and a recent experimental study showed that vitamin D deficiency promoted AAA growth and rupture in mice [139], but further clinical studies are needed to resolve whether a putative antioxidant effect of vitamin D would benefit AAA patients [140].

### 4.8. The Role of Diabetes Mellitus

Although diabetes mellitus is a major risk factor for occlusive CVDs, type II diabetes is associated negatively with AAA. Multiple meta-analyses of numerous large-scale epidemiological and clinical studies show that the risk of developing AAA as a diabetic patient is about half that of a nondiabetic [141,142,143]. Furthermore, diabetics show lower AAA growth rates than nondiabetic AAA patients. It is estimated that type II diabetes slows the annual growth of an AAA by 0.51–0.60 mm or about 25% [28,30,144]. A meta-analysis investigating rupture risk found that diabetes was associated with significantly lower rates of rupture [145]. However, diabetic patients show increased mortality after AAA repair and lower survival rates over 2–5 years [141], which may be an indication of the higher overall cardiovascular burden of diabetic patients [146].

This curious relationship between diabetes and AAA may be mediated by the hyperglycemic environment and thickened aortic wall prevalent in diabetics exerting protective effects both biochemically and mechanically [147]. Experimental studies support a possible attenuation effect of hyperglycemia on AAA development and growth [148,149]. In recent years, evidence has emerged that indicates the beneficial effect of diabetes on AAA may in fact be mediated by the antidiabetic medication metformin [150,151,152]. This aspect will be explored further in the chapter on pharmacological approaches to limit AAA growth.

## 5. Diagnosis and Management

Most intact AAAs are diagnosed incidentally when patients undergo imaging of the abdominal region for an unrelated condition. Besides ultrasonography and computed tomography, imaging modalities that may detect an AAA are diverse and include spinal imaging and echocardiography [153,154], though analyses of sensitivity and specificity for these modalities are scarce [155,156]. In some countries, systematic screening programs by ultrasonography are in place [157]. After initial diagnosis, all patients should be referred to a vascular surgeon to determine further proceedings [158].

### 5.1. Screening Programs

Long-term follow-up studies of large randomized controlled trials (RCTs) from the 1990s revealed that screening all 65-year-old men by one-time ultrasonography reduces AAA-related mortality by 42–66% [159,160], with corresponding ORs in favor of screening of 0.53–0.60 in meta-analyses [161,162]. Nationwide screening programs have since been implemented in several countries such as the USA, UK, and Sweden [157]. Recent results from these programs and large population-based trials confirm the benefits of screening men with regard to AAA-specific mortality, though there are conflicting results regarding all-cause mortality [161,162]. It is estimated that 667 men need to be screened in order to prevent one premature AAA-related death [163]. Based on this calculation, screening of 65-year-old men for AAA is considered cost-effective and was implemented as a preventive health measure in Sweden [163]. This strategy has been proposed to remain cost-effective, even if AAA rates should drop by half [164]. Yet, the declining prevalence of disease is one of the major arguments for the decision of other governments against nationwide screening programs.

Despite possible overdiagnoses, the psychological consequences of an AAA diagnosis, and decreasing incidence and prevalence rates, the benefits of screening men are commonly considered to outweigh the drawbacks [164,165,166]. Analyses of cost-effectiveness of screening for men also raise the question of targeted screenings for other at-risk groups. It is estimated that screening only male smokers between the ages of 65 and 75 leads to an AAA detection rate of about 30% in the population aged 50–84 [49]. While most current guidelines do not recommend population screenings for women, again mainly due to low prevalence and cost-effectiveness considerations [10,157], Canada has recently challenged this notion and proposed one-time screening by ultrasonography for women of advanced age with a history of smoking or cardiovascular disease [167]. No assessments of feasibility and cost-effectiveness have yet been performed for other candidate groups, such as patients with other peripheral aneurysms [168], cardiovascular diseases [113,169], or a subaneurysmal aortic dilation (2.5–2.9 cm in diameter) [32,170]. Some authors aim to develop scoring tools for possible refinement of AAA screening strategies [171,172].

### 5.2. Imaging Techniques

Ultrasonography (US) and computed tomography angiography (CTA) are first-line imaging tools for detection and management of AAAs. CTA is the gold standard for the diagnosis of AAA rupture, therapeutic decision making, and treatment planning, as well as post-surgical assessment and follow-up [173,174]. It provides detailed anatomical information on the entire aorta and its adjacent vessels, allowing assessment of the extent of the AAA, and of possible acute and chronic comorbid pathologies and, thus, exact planning of surgical intervention [175]. Due to its wide availability, rapid image acquisition, and lower radiation burden, CTA has virtually rendered conventional invasive angiography for the evaluation of AAA obsolete.

Duplex US is the recommended modality for screening and diagnosis of asymptomatic patients [158,176], as it allows safe, noninvasive, and fast detection of AAAs with high sensitivity and specificity [7,177]. It can also be used in emergency settings for rapid assessment of symptomatic patients [178], but emergency conditions with possible active bleeding after rupture or endoleaks after endovascular aneurysm repair (EVAR) usually warrant an additional CTA due to inherent methodological limitations of US scans. For instance, adjacent vessels may be difficult to assess, and imaging methodology influences diameter measurements [179,180,181]. With both US and CTA, standardization of methodology and reporting standards are crucial to obtain reliable results and to reduce intra- and interobserver variability [182,183].

Magnetic resonance angiography (MRA) shares many of the advantages of CTA imaging, with the added plus of not exposing the patient to radiation or requiring iodinated contrast agents. These factors make MRA a viable alternative for patients with certain allergies or renal insufficiency [183,184]. However, contraindications for MR such as claustrophobia and some metal implants have to be considered, and MR imaging is not suitable in emergency situations, such as impending or suspected rupture [185].

Molecular imaging by positron emission tomography–computed tomography (PET–CT) is not routinely used in standard care for AAA, but may add valuable information for diagnosis and follow-up of specific pathologies, such as inflammatory or mycotic AAA and infected stent grafts [186,187]. In vivo visualization of functional activity by uptake of radiotracers enables quantification of metabolic activity of cells and, thus, may facilitate further understanding of AAA pathogenesis and, in the future, possibly provide a novel diagnostic tool for clinical risk stratification [188,189].

### 5.3. Management of Small AAAs

As no pharmacological therapy has been established yet to slow AAA growth or prevent rupture, surgical repair is the only curative treatment for AAAs. Thus, disease management requires careful assessment and gauging of the surgical risk versus the risk of aneurysm rupture. For as long as the risk of elective surgical repair exceeds the risk of rupture, conservative management, i.e., watchful waiting while following the current guidelines on cardiovascular disease control by best medical care, is indicated [35]. The safety of this course of action has been determined by several RCTs and large studies comparing early elective repair to surveillance [190,191,192,193]. For patients with small AAAs, early elective repair offers no advantage, and surveillance, coupled with best medical care and lifestyle modification, is considered safe and cost-effective [194].

Regular monitoring of aneurysm growth and symptoms is recommended at intervals between three years for aneurysms of 3.0–3.9 cm diameter, annually for aneurysms of 4.0–4.9 cm, and every 3–6 months for AAAs with a diameter ≥5.0 cm [158,176], but may be adapted according to individual patient factors, such as fast aneurysm growth or high peak wall stress [35,195]. Insufficient monitoring of incidentally detected AAAs is associated with increased mortality [196,197].

#### 5.3.1. Control of Cardiovascular Risk

While aneurysm diameter is an important measure of disease progression, adequate control of cardiovascular risk factors is crucial to improve outcomes [198,199]. Control of cardiovascular risk factors can be accomplished by lifestyle modifications and auxiliary medication to manage aggravating conditions, such as hypertension or dyslipidemia.

Cessation of smoking is a key component of CVD prevention or reduction, and additionally, significantly lowers post-surgical pulmonary complications if achieved more than eight weeks before elective AAA repair versus quitting short-term [200,201]. A well-balanced diet reduces CVD events and improves outcomes, and was suggested to be even more effective in reducing obesity compared to exercising [202,203]. Nevertheless, physical activity can alleviate the CVD-associated health risks of excess weight and improve surgical outcomes [133,204,205].

When indicated, pharmaceutical management of cardiovascular risk factors is recommended for all AAA patients, unless contraindicated [158,176]. In line with CVD risk assessment, statins are applied to reduce LDL cholesterol to <110 mg/dL for low risk, to <70 mg/dL for intermediate risk, and to <55 mg/dL for high risk [206,207]. Systolic blood pressure should be maintained below 140 mmHg. The choice of specific antihypertensive drug depends on individual comorbidities and tolerability, though diuretics may be less beneficial than other antihypertensive drug classes [118]. Antiplatelet agents are another constituent to prevent cardiovascular events in AAA patients. A UK study examining medication regimens of AAA patients showed that patients taking statins, antiplatelet therapy, or antihypertensive medication had significantly improved five-year survival rates (68% vs 42%, 64% vs 40%, and 62% vs 39%, respectively) [118].

#### 5.3.2. Pharmacological Approaches to Limit AAA Growth

A wide range of drug classes has been investigated for possible benefits in AAA treatment, as we have previously reviewed [208], but as of yet, no medical therapy has been found sufficiently effective in reducing AAA growth or in preventing rupture to be implemented in the treatment guidelines [158,209,210].

Betablockers were the first drug class to be examined in the context of AAA growth reduction. While initial results seemed encouraging, larger studies and RCTs found low tolerability of the drug regimen and no beneficial effect of propranolol treatment on AAA expansion [211,212,213]. For another class of antihypertensive drugs, inhibitors of angiotensin-converting enzyme (ACE), all but one study revealed no effect on AAA expansion either [214,215,216,217]. The deviating result originated from a prospective cohort study that intriguingly found increased growth rates in patients taking ACE inhibitors [218].

Antibiotics were thought to be another promising drug class, as chlamydia pneumoniae has been implicated in AAA pathogenesis [219,220]. Although doxycycline was shown to significantly reduce plasma markers of proteolytic activity [221,222], clinical studies obtained contradictory results and a recent RCT reported no decline in AAA growth [223,224,225]. Results for the macrolide antibiotics roxithromycin and azithromycin were similarly discouraging [226,227,228].

The influence of statins on AAA growth has been discussed controversially, as clinical studies were not able to consistently reproduce the promising results of experimental investigations but showed heterogeneity of outcome. Several meta-analyses tentatively support the ability of statins to reduce AAA growth [229,230,231], while others err on the side of caution [232]. Furthermore, ethical considerations and guidelines limit performing RCTs on statins, as they significantly increase 5-year survival rates in AAA patients [118,233].

Several other drug classes, such as nonsteroidal anti-inflammatory drugs [234], mast cell inhibitors [235], calcium channel blockers, diuretics, and angiotensin II receptor blockers [213,236] have been investigated clinically after showing promise in experimental studies, but none of these studies were able to show a reduction in AAA growth. Antiplatelet therapy by acetylsalicylic acid or ticagrelor seemed another auspicious treatment approach [237,238,239], but only one subanalysis of a small azithromycin RCT was able to detect a significant effect of acetylsalicylic acid on growth rate [228]. Currently, several RCTs are ongoing with hopes of finding a medical treatment for AAA [240,241,242,243].

An avenue of research that raises hope for successful AAA treatment in the future is a well-established antidiabetic medication. The previously observed protective effect of diabetes mellitus on AAA progression may not actually be inherent to the disease, but rather attributable to concurrent treatment with metformin [151,152,244]. Experimental studies were investigating a range of medications such as thiazolidinediones and dipeptidyl peptidase-4 inhibitors [245,246], but metformin seems to be the only drug consistently associated with reduced AAA growth in clinical studies [247]. Currently, two ongoing clinical trials [242,243] and a shortly upcoming study [248] are investigating whether metformin reduces AAA progression in nondiabetic patients, one of them at the Division of Vascular Surgery of the Medical University of Vienna [242].

Future perspectives of pharmacological approaches to limit AAA growth also include nucleic acid drugs that target mRNAs, microRNAs, or transcription factors in a sequence-specific manner to interfere with key molecules in AAA pathogenesis [249].

### 5.4. Surgical Treatment

Surgical repair is the only curative treatment for AAAs and is indicated when the risk of rupture exceeds the surgical risk. Nowadays, about 85% of AAA repairs are performed electively for intact aneurysms, though there are significant regional variations [250]. Naturally, a ruptured AAA is a surgical emergency that requires immediate repair, with a significant mortality of up to 85% [251,252].

Based on data from several RCTs comparing early elective repair to surveillance [194,253], current guidelines recommend elective AAA repair for asymptomatic fusiform AAAs at an aneurysm diameter of 5.5 cm in men and 5.0 cm in women, if the surgical risk is acceptable [158,176]. Some authors suggest reevaluation of the threshold for surgical repair for women in future guidelines [254], as the current cutoff diameters signify a larger relative rupture risk for women than for men [255,256]. Earlier elective repair should also be considered for saccular AAAs [257,258]. In the case of a rapid expansion rate of >10 mm/year or incident symptoms referable to the aneurysm, additional imaging for confirmation and then fast-track referral to a vascular surgeon is recommended, even at small diameters [259,260].

#### 5.4.1. Open Surgical Repair

Open AAA repair aims to replace the aneurysmal wall by a synthetic vascular graft. The surgery involves a laparotomy, usually by midline incision, and transperitoneal or retroperitoneal exposure of the proximal abdominal aorta just below the renal arteries [261]. Depending on the aneurysm’s morphology and size, either tube-shaped or bifurcated grafts can be used. After aortic cross clamping under full heparinization [262], the proximal end-to-end anastomosis is performed as close as possible to the renal arteries to counteract development of another aneurysm in the remaining infrarenal aortic portion [263]. The exact location of the distal dissection site depends on the individual’s anatomy, the AAA’s extent, and concomitant conditions, such as local atherosclerotic burden or an aneurysm of the iliac arteries. Finally, the aneurysm sac is closed over the graft.

Perioperative complications of open surgical repair (OSR) are mainly of cardiac, pulmonary, or renal nature, such as myocardial infarction, pneumonia, or renal insufficiency [264]. Postoperative wound complications can affect a patient’s recovery severely [265], and midline laparotomy incisions are more prone to incisional hernias [266,267]. Long-term complications include graft infections, formation of secondary aorto-enteric fistulas, graft limb occlusion, or formation of a para-anastomotic aneurysm [268,269].

#### 5.4.2. Endovascular Aneurysm Repair

EVAR does not aim to replace the aneurysmal sac, but excludes it from systemic circulation by minimally invasive implantation of a stent graft [270]. The stent graft is delivered to the abdominal aorta through the femoral artery, either by a percutaneous approach or by a surgical cut-down [271]. After confirmation of the exact vessel measurements by digital subtraction angiography, the delivery system is inserted over a stiff guidewire and then deployed and ballooned to expand and attach the stent graft to the aortic wall. Fixation and sealing of the stent grafts require certain morphological attributes, such as low angulation of the neck, and a certain length and diameter of the proximal and distal fixation sites [272]. Enhanced modular, fenestrated, bifurcated, or branched stent graft designs facilitate precise tailoring to individual anatomy, and allow for anatomical deviations such as accessory vessels, inadequate landing zones, or concomitant conditions [273,274].

Inadequate sizing or improper placement of the stent graft can cause various complications such as endoleaks from inadequate sealing or kinking of the stent graft with subsequent limb occlusion [275,276]. An endoleak is defined as persistent blood flow inside the aneurysm sac, resulting in a rise in pressure, renewed aneurysm growth, and eventual rupture. The various types of endoleaks are the most common complication of EVAR and often require reintervention [277]. Other complications of EVAR include problems related to the access site, especially when the iliac arteries are small, calcified or severely tortuous [271], and a systemic inflammatory response termed ‘post-implantation syndrome’ in the early postoperative phase [278]. Complex endovascular aortic repair should only be advised at tertiary university hospitals, due to the necessary case numbers for experience in both complex EVAR and OSR, in case of complication management [273,274]. 

Another novel concept, termed endovascular aneurysm sealing, relies on complete sealing of the aneurysm sac by way of surrounding the stent graft with polymer-filled endobags [279], but long-term durability is questionable [280,281,282].

#### 5.4.3. Comparison of Surgical Methods

The choice of surgical technique depends on the aneurysm’s morphology and on patient characteristics, such as comorbidities and functional capacity [283,284]. Another consideration is the patient’s ability and willingness to adhere to necessary follow-up surveillance [285,286], as EVAR requires life-long surveillance with regular imaging, owing to the risk of late-onset endoleak, stent graft migration, or infection [287].

Exact assessment of anatomical and morphological attributes by pre-operative CT or MR angiography is crucial to ensure the best possible outcome [288]. With both OSR and EVAR, due care has to be exercised with regard to branch vessels to ensure adequate blood flow in renal, superior mesenteric, lumbar, and internal iliac supply zones to prevent postoperative sexual dysfunction and ischemic complications caused by inadequate pelvic circulation [289,290,291].

While initially intended for patients deemed unfit for OSR, EVAR has since become the method of choice if technically feasible, and nowadays, more than three quarters of elective AAA repairs are performed endovascularly [158,176]. With this dynamic change in the ratio of OSR to EVAR, a minimum of >30 cases annually per center is required in both techniques to achieve the required expertise [158,176]. A major advantage of EVAR over OSR is that it can be performed under local or epidural as well as general anesthesia, rendering it a possibility for patients with severe cardiac and pulmonary comorbidities [292,293]. However, the endovascular approach does not offer any long-term survival benefits in patients considered too physically frail for open AAA repair [294]. In patients currently unfit for elective surgery, optimization of functional capacity and overall cardiovascular health should be pursued before reassessment of surgical eligibility [294,295].

Open AAA repair is indicated for patients whose vessels do not meet the requirements of endovascular repair, for instance due to short landing zones or excessive thrombus formation [296]. OSR may also be required for treatment of certain EVAR-specific complications such as persistent endoleaks or aneurysm sac growth, as well as for patients with an inflammatory aneurysm or an infected graft.

#### 5.4.4. Outcome of Elective AAA Repair

After the advent of EVAR in the 1990s, several RCTs aimed to evaluate outcomes of EVAR versus OSR for elective AAA repairs. A 2014 meta-analysis of these trials and more recent studies found that EVAR has better short-term outcomes than OSR in both morbidity and mortality [270]. Compared to OSR, cardiopulmonary complications are significantly lower in EVAR due to its minimally invasive approach. It is not surprising, therefore, that patients with EVAR benefit from a faster recovery and shorter length of hospital stay. EVAR’s early benefits are offset against lower durability. Due to graft-related complications, the reintervention rates and AAA-related mortality are significantly higher for EVAR compared to OSR [264]. In the long term, outcomes balance out, and long-term return of functional level and health-related quality of life are similar between OSR and EVAR for elective AAA repair [297]. Despite the need for continuous follow-up and high rate of reinterventions, EVAR remains cost-effective when compared to OSR [298].

EVAR is associated with lower 30-day mortality (1.4% for EVAR, 4.2% for OSR) and a higher survival rate for up to one year after the repair [264,270,299]. However, this survival advantage is lost around the 2–4-year mark of follow-up, and long-term all-cause survival is similar for EVAR and OSR. Median survival after elective AAA repair is about 9 years, with cardiovascular and pulmonary diseases constituting the main causes of death [117,264,300]. Adequate management of cardiovascular risk factors improves long-term survival after AAA repair [301].

Besides advanced age and presence of cardiac, pulmonary, and renal comorbidities, frailty and preoperative functional status are predictors of postoperative morbidity and mortality [302,303,304]. Female sex, aneurysm diameter, and smoking habits are also associated with poor long-term survival. The worse outcomes for women may be due to a higher frequency of disadvantageous neck anatomy, but these anatomical differences do not seem to account for all differences in outcomes between men and women [56,305]. Increased cancer mortality rates in EVAR patients when compared to OSR have also raised concerns about long-term radiation exposure during EVAR follow-up [306].

#### 5.4.5. Management of the Ruptured AAA

A ruptured AAA with acute hemorrhage into the intra- or retroperitoneal space is a surgical emergency. Depending on the hemodynamic situation, an immediate CTA, or alternatively, an intraoperative angiography is indicated to confirm the diagnosis and evaluate anatomical suitability for EVAR [307,308]. Both OSR and EVAR can be used for rAAA repair, with EVAR being the method of choice if anatomically feasible. EVAR and open surgery for rAAA have comparable morbidity rates and show no difference in cardiac or respiratory failure [251]. Reintervention rates are similar, as well, and EVAR is consistently associated with faster discharge and a gain in quality-adjusted life years, rendering it cost-effective [309].

As with elective AAA repairs, use of EVAR for rAAA repair has increased massively in the past two decades [251,252]. Analyses of earlier versus later cohorts show that outcomes have improved in recent years for both EVAR and OSR [310,311]. High mortality rates of unsuccessful EVAR for rAAA suggest that anatomical suitability and not hemodynamic condition should be the pivotal factor in choosing the surgical method for rAAA repair [251,312,313].

## 6. Pathogenesis

The development of an AAA is an intricate process of several pathomechanisms and is still not fully understood. Ignited by a possibly diverse initial trigger, a destructive process of oxidative stress, apoptosis of vascular smooth muscle cells (VSMCs), and proteolytic fragmentation of the extracellular matrix (ECM) are set in motion, potentiated by an inflammatory immune response. These key processes perturb the equilibrium between regenerative and degrading processes that normally ensures physiological tissue remodeling and injury repair. Now malfunctioning, the aortic wall becomes progressively eroded and weakened, dilates, and finally ruptures when it can no longer withstand the hemodynamic forces placed upon it.

### 6.1. Intraluminal Thrombosis and Biomechanical Aspects

The infrarenal portion of the aorta is especially prone to aneurysms, due to hemodynamic and mechanical characteristics. Due to the blood stream’s impact on the iliac bifurcation, pressure-reflective waves within the blood are common in the infrarenal aorta. These disturbances lead to a higher number of collisions of circulating cells amongst each other and against the aortic wall. Endothelial injuries and atherosclerotic lesions often form at such locations, and the high wall stress and strain caused by the aortic blood stream likely contribute to the persistence of the endothelial injury at this location [314,315].

Historically, AAA was thought to be a particular, localized form of atherosclerosis, as most AAAs present with cholesterol and calcium deposits. The exact nature of the relationship between atherosclerosis and AAA is still a matter of discussion [106]. In both diseases, an atherosclerotic plaque may form on the basis of an intimal lesion and subsequently replace the subendothelium. In occlusive atherosclerotic diseases, inward remodeling of the aortic wall usually leads to a decrease in vessel lumen until either an erosion- or rupture-triggered thrombus itself or an embolus cause occlusion of the vessel to a hemodynamically relevant degree. Different theories have been proposed as to the role of the gradually forming intraluminal thrombus (ILT) in about 75% of AAA cases, such as a propensity of the aortic wall of AAA patients towards outwards remodeling in an attempt to maintain the vessel lumen [41,316,317].

Whether causally associated or a subsequent byproduct, the ILT provides the aneurysmal wall with a certain biomechanical protection against wall stress [318,319]. Permeation of the ILT with blood or contrast medium may announce the impending failure of the ILT as a protective layer. This distinguishing mark is visible as a ‘crescent sign’ in CTA [320] and is associated with AAA rupture [174,321,322]. However, clinical data support the notion that the ILT’s active role in disruption of the wall’s integrity exceeds its biomechanical protection. Indeed, presence of an ILT is associated with an increased rupture risk, especially in patients with small AAAs and when the thrombus encompasses the whole circumference of the aneurysm [45,323,324].

The ILT in AAA is a biologically active, dynamic component that contributes to the pivotal destruction and inflammation of the aortic wall’s medial and adventitial layers [325,326,327]. The presence of an ILT in an AAA is associated with reduced thickness of the artery wall, greater elastolysis, lower VSMC content in the medial layer, and a higher level of inflammatory immune response in the adventitia [328]. Clinical data also show that the presence of an ILT is associated with faster AAA growth [329], which may be due to its biological activity [330,331], rather than size or volume [332,333,334].

The blood stream constantly replenishes the ILT’s luminal side with fibrinogen and circulating cellular elements, such as platelets, erythrocytes, and immune cells [335]. The ILT entraps these cells, which release oxidative enzymes, proteases and proinflammatory cytokines [336,337,338]. Due to the high pressure gradient between the aortal blood flow and the interstitial tissue, the cells and released molecules are pushed outward towards the abluminal side through a canalicular system pervading the ILT [325]. Thus, the ILT contributes to the generation of a toxic environment that contributes to the decay and inflammation of the subjacent medial and adventitial layers of the aortic wall, as we have previously highlighted [339].

### 6.2. Oxidative Stress and VSMC Apoptosis

As mentioned above, the ILT accumulates blood cells, i.e., platelets, erythrocytes, and leukocytes. The adverse environment increases reactivity of entrapped cells, which in turn boost oxidative stress by mediating production of reactive nitrogen and oxygen species (ROS) [324,340,341]. These excess oxygen- and nitrogen-derived free radicals then activate proteolytic enzymes, trigger degradation processes, and induce apoptosis of VSMCs and mesenchymal progenitor cells [324,342]. This not only destabilizes the medial layer of the aortic wall, but also impedes its matrix-producing and -repairing capacities. Proinflammatory signals further stimulate VSMC apoptosis [343,344].

Excessive cell death and dedifferentiation (‘phenotypic switch’) of contractile VSMCs is characteristic of the aneurysmal aortic wall [345,346]. Loss of VSMCs puts additional hemodynamic stress on the weakened wall, which responds by stimulating angiogenesis in the medial layer [347,348]. Furthermore, the ILT in place of the endothelium causes localized hypoxia in the underlying AAA wall, which is an additional trigger for angiogenesis. However, medial neovascularization further instigates the destructive cycle because it facilitates infiltration of circulating inflammatory cells [349,350].

Clinical data support the importance of oxidative stress in AAA pathogenesis [351]. Excess levels of ROS have been detected in the blood of AAA patients and in AAA wall tissue, but not in the adjacent nonaneurysmal aorta or in healthy individuals [340,352]. These ROS stem from a variety of oxidative enzymes shown to be upregulated in expression and activity in aneurysmal aortic walls [353,354]. In mice, loss of antioxidants was found to increase AAA incidence and rupture rate [355]. Correspondingly, experimental activation of antioxidant enzymes, such as catalase, reduces AAA formation in mice [356,357,358], but a benefit of ROS-suppression in humans has not been shown yet [343].

### 6.3. Proteolysis

The destruction of the three-dimensional ECM network of the medial layer of the aortic wall is considered a key feature of AAA pathogenesis. Excessive dismantling of structure-bearing components reduces the wall’s ability to withstand the hemodynamic load and leads to dilation of the aorta [359,360]. The physiological tissue remodeling process becomes unbalanced and tilts towards an overactivity of proteases that disintegrate the fibrillar ECM components, such as collagen and elastin [361,362,363]. Protease substrates also include cell adhesion molecules, thereby promoting VSMC detachment and apoptosis [344,364,365]. In addition, proteolytic enzymes augment AAA pathogenesis by activating ECM-contained, latent matrix metalloproteinases (MMPs), and by mediating inflammation and angiogenesis [366,367,368,369]. In turn, inflammatory conditions and oxidative stress boost production and activation of proteases, creating a destructive circle [370,371].

Several protease families have been implicated in the pathogenesis of AAAs. Clinical data show increased levels of MMPs, especially MMP-9 and MMP-2, cathepsins, and neutrophil elastase in blood and tissue samples of the aneurysmal wall of AAA patients, while their antagonists are reduced [122,372,373]. Population-based studies showed that high cathepsin levels are associated with higher AAA risk and a larger diameter [374,375]. MMP-9 plasma levels were reported to decrease after AAA repair [376,377]. In rodent studies, cathepsin deficiency was found to reduce aneurysm severity [378,379,380], and both MMP-2 and MMP-9 knockout mice were protected from AAA development compared to wild-type mice [381,382]. However, despite their central role in AAA development, unselective MMP inhibition by doxycycline has been unsuccessful in attenuating AAA growth in humans [223,224], illustrating that complex interactions and multiple mechanisms are at play.

### 6.4. Inflammation

Aneurysmal aortic wall tissue is characterized by infiltration of both innate and adaptive immune cells. At the luminal side, the ILT recruits circulating leukocytes, which then migrate towards the media. Diapedesis is facilitated by the medial layer’s angiogenesis, hemodynamic factors, as well as by activation of the complement system [383,384,385]. Other entry points for immune cells to infiltrate the aortic wall are periadventitial lymph nodes and adventitial vasa vasorum. Development of tertiary lymphoid organs in the adventitia by a concerted collaboration of B-lymphocytes, follicular dendritic cells, and CD4+ T-helper-cells has been described [386,387]. Ectopic adipocytes in perivascular tissue promote recruitment and activity of immune cells by releasing proinflammatory cytokines, such as tumor necrosis factor alpha (TNF-α), IL-6, IL-8 [388,389], and may also mediate an autoimmune response [390].

Cytokines released by both innate and adaptive immune cells contribute to the inflammatory environment in the AAA wall. High levels of adventitial CD4+ T-helper-cells and macrophages mediate recruitment and activation of inflammatory cells, establishing a vicious circle of chronic inflammation of the aortic wall, while also boosting proteolysis and oxidative tissue injury [341,391]. Circulating levels of Th1- and Th2-secreted cytokines such as IFN-γ, TNF-α, IL-4, and IL-22 are consistently elevated in AAA patients [392,393]. IFN-γ and IL-17 secreted by CD8+ T-cells and Th17 CD4+ T-cells have also been implicated in AAA pathogenesis [394,395,396]. B-cell-derived immunoglobulins such as IgG4 and IgE drive macrophage and mast cell activation and degranulation [397,398]. Regulatory CD4+ T-cells have a protective function by releasing anti-inflammatory cytokines such as IL-10 and TGF-β [399,400,401], but both regulatory CD4+ cells and their mediators are reduced in AAA patients [402].

A prospective clinical study using enhanced MR imaging has shown that the degree of aortic wall inflammation predicts AAA growth and rupture [331]. The crucial role of inflammation is supported by a number of experimental rodent studies showing that depletion of immune cells, in particular myeloid cells, diminishes or abolishes AAA growth [41,403].

### 6.5. Myeloid cells in AAA

#### 6.5.1. Monocytes and Macrophages in AAA

Monocytes and macrophages have been consistently detected in AAA tissue of both rodent disease models and humans [404,405,406,407]. These immune cells of myeloid origin infiltrate early in aneurysm development, and predominantly accumulate in the inner adventitial layer of the aneurysmal aortic wall [349]. Monocyte and macrophage chemoattractants and activation parameters (e.g., chemokine ligand [CCL] 2, also referred to as monocyte chemoattractant protein 1, MCP-1 [393,408], CCL3 alias macrophage inflammatory protein-1a, MIP-1a [408]), receptors (e.g., chemokine C-X-C receptor 4, CXCR4 [409], triggering receptor expressed on myeloid cells-1, TREM-1 [410]) have been shown to be significantly increased in rodent models, AAA patient plasma, and aneurysmal wall tissue. Blocking or depleting the involved ligands or pertaining receptors inhibits AAA formation and progression in animal models (e.g., C-C chemokine receptor type 2, CCR2 [404], CXCR4 [409], CCL5 [411]).

Monocytes and macrophages contribute to AAA pathogenesis in various ways, depending on the functional characteristics of their subtype. Monocytes can be classified into distinct subsets [412,413] that assume different roles in the elimination of pathogens and maintenance of vessel integrity [414,415,416]. For instance, CD16-expressing monocytes with tissue-remodeling and proangiogenic features are associated with AAA diameter, and have been reported to be significantly elevated in AAA patient plasma [408,417,418]. Similarly, macrophages exhibit two distinct phenotypes that have opposing functions in mediating inflammatory processes [419]. An imbalance between the two macrophage phenotypes can create either a chronic inflammatory milieu or impairment of wound healing and tissue homeostasis [420]. Although analyses of AAA tissue have not yielded conclusive results to such a disproportion [407,421], rodent studies indicate that proinflammatory, destructive M1 macrophages may predominate during early stages of AAA development [422]. The proteinases and proinflammatory mediators released by M1 macrophages in close collaboration with T-cells contribute to aneurysmal wall degradation [407,423]. New data suggest that the M2 macrophage may not be entirely protective [424], as experimental rodent studies investigating macrophage polarization from M1 to M2 have claimed that M2 polarization can either suppress or attenuate AAA development [425,426,427].

#### 6.5.2. Neutrophils in AAA

Neutrophils pose a crucial first line of defense in the innate immune system, as they are the most numerous type of granulocytes (or ‘polymorphonuclear leukocytes’) and can employ several effector mechanisms to eliminate pathogens [428]. As we have recently reviewed [429,430], neutrophils are recruited to the aneurysm site by various chemotactic factors, such as IL-8, platelet-derived factors or complement components. These mediators are mainly released from the luminal ILT and have been shown to be increased in the blood of AAA patients [337,393,431]. In rodent models, blockage or depletion of neutrophil chemotactic factors can impede or completely abolish AAA development by suspending neutrophil recruitment [383,432]. For instance, dipeptidyl peptidase I (DPPI) regulates the activity of neutrophil-secreted proteases, but also mediates neutrophil recruitment to the aneurysm site. Functional deficiency of DDPI has been shown to cause impaired local production of a neutrophil chemotactic factor and thereby protect mice from elastase-induced AAA formation [432].

Elevated levels of activated neutrophils have been detected in AAA wall tissue [337,349], as well as in AAA patient plasma in comparison to healthy controls, even after adjustment for known confounders such as smoking [408,433]. Activation of neutrophils induces degranulation, i.e., the release of cytotoxic contents of the neutrophil’s cytoplasmic granules into the phagosome or into the extracellular space [434]. Besides bactericidal peptides (e.g., lactoferrin), defensins, and proteinases (e.g., neutrophil elastase, collagenase, cathepsins), neutrophil granules release myeloperoxidase (MPO) and nicotinamide adenine dinucleotide phosphate oxidase (NADPH oxidase or NOX). MPO and NOX enzymes are major sources of ROS and have been detected at high levels in AAA tissue and AAA patient plasma, while antioxidants such as catalase are reduced [351,352,435].

ECM degradation is facilitated by several neutrophil-mediated mechanisms. MPO has been shown to inactivate tissue inhibitors of MMPs, thereby increasing MMP activity [436]. Furthermore, neutrophils release neutrophil-gelatinase-associated lipocalin (NGAL), which binds MMP-9 and inhibits its degradation [338]. High concentrations of NGAL-MMP-9-complexes are present especially in the luminal ILT [335,337], and are higher in aneurysmal aortic wall samples compared to healthy aortas [437]. Neutrophil-secreted proteases directly cleave stabilizing ECM components, but can also activate MMPs [366,438,439]. Fragments of the dismantled elastin and collagen fibers then promote inflammation further by serving as chemotactic factors themselves [440,441,442]. As mentioned above, DPPI is not only necessary for activation of neutrophil-secreted proteases, but also vitally mediates inflammation of the aneurysmal aortic wall [432].

A 2005 study showed neutrophil-depleted mice to be protected from AAA formation [403]. However, contrary to the expected, this was only in part mediated by a decrease in MMP activity, suggesting that neutrophils critically contribute to AAA pathogenesis by another, independent mechanism. A novel effector mechanism of neutrophils, the formation of neutrophil extracellular traps (or ‘NETs’), has since been implicated in AAA pathogenesis [443,444,445], which is the current focus of our research efforts [430,446]. 

## 7. Conclusions

Even though world-wide changes in smoking habits and other lifestyle factors have contributed to declining incidence and mortality rates of AAA, its prevalence and high death rate upon vessel rupture remain a subject of concern. Progress has been made in unraveling the multifactorial process of pathogenesis, in particular the components of chronic inflammation. This has led to promising candidates for conservative treatment such as metformin, an antidiabetes drug with pleiotropic anti-inflammatory effects, which is currently being tested in several prospective clinical trials to prevent AAA progression. Yet to date, surgical repair remains the only curative approach, but is associated with a considerable morbidity and mortality. The rise in endovascular versus open surgical interventions has revealed the challenge of establishing sufficient institutional expertise in both procedures in order to appropriately address patient needs.

## Data Availability

Not applicable.

## References

[1] Johnston K., Rutherford R.B., Tilson M., Shah D.M., Hollier L., Stanley J.C. (1991). Suggested Standards for Reporting on Arterial Aneurysms. Subcommittee on Reporting Standards for Arterial Aneurysms, Ad Hoc Committee on Reporting Standards, Society for Vascular Surgery and North American Chapter, International Society for Cardiovascular Surgery. J. Vasc. Surg..

[2] Matsumoto T. (2019). Anatomy and Physiology for the Abdominal Aortic Aneurysm Repair. Ann. Vasc. Dis..

[3] Takayama T., Yamanouchi D. (2013). Aneurysmal Disease: The Abdominal Aorta. Surg. Clin. North Am..

[4] Golledge J., Muller J., Daugherty A., Norman P. (2006). Abdominal Aortic Aneurysm: Pathogenesis and implications for management. Arter. Thromb. Vasc. Biol..

[5] Lederle F.A., Johnson G.R., Wilson S.E., Chute E.P., Littooy F.N., Bandyk D.F., Krupski W.C., Barone G.W., Acher C.W., Ballard D.J. (1997). Prevalence and Associations of Abdominal Aortic Aneurysm Detected through Screening. Aneurysm Detection and Management (ADAM) Veterans Affairs Cooperative Study Group. Ann. Intern. Med..

[6] Rogers I.S., Massaro J., Truong Q.A., Mahabadi A.A., Kriegel M.F., Fox C.S., Thanassoulis G., Isselbacher E.M., Hoffmann U., O’Donnell C.J. (2013). Distribution, Determinants, and Normal Reference Values of Thoracic and Abdominal Aortic Diameters by Computed Tomography (from the Framingham Heart Study). Am. J. Cardiol..

[7] Lindholt J., Vammen S., Juul S., Henneberg E., Fasting H. (1999). The Validity of Ultrasonographic Scanning as Screening Method for Abdominal Aortic Aneurysm. Eur. J. Vasc. Endovasc. Surg..

[8] Steinberg C.R., Archer M., Steinberg I. (1965). Measurement of the Abdominal Aorta after Intravenous Aortography in Health and Arteriosclerotic Peripheral Vascular Disease. Am. J. Roentgenol. Radium Ther. Nucl. Med..

[9] Li K., Zhang K., Li T., Zhai S. (2018). Primary results of abdominal aortic aneurysm screening in the at-risk residents in middle China. BMC Cardiovasc. Disord..

[10] Sweeting M.J., Masconi K.L., Jones E., Ulug P., Glover M.J., Michaels J.A., Bown M.J., Powell J.T., Thompson S.G. (2018). Analysis of clinical benefit, harms, and cost-effectiveness of screening women for abdominal aortic aneurysm. Lancet.

[11] Tang W., Yao L., Roetker N.S., Alonso A., Lutsey P.L., Steenson C.C., Lederle F.A., Hunter D.W., Bengtson L.G., Guan W. (2016). Lifetime Risk and Risk Factors for Abdominal Aortic Aneurysm in a 24-Year Prospective Study: The ARIC Study (Atherosclerosis Risk in Communities). Arter. Thromb. Vasc. Biol..

[12] Li X., Zhao G., Zhang J., Duan Z., Xin S. (2013). Prevalence and Trends of the Abdominal Aortic Aneurysms Epidemic in General Population—A Meta-Analysis. PLoS ONE.

[13] Savji N., Rockman C., Skolnick A.H., Guo Y., Adelman M., Riles T., Berger J.S. (2013). Association between Advanced Age and Vascular Disease in Different Arterial Territories: A Population Database of over 3.6 Million Subjects. J. Am. Coll. Cardiol..

[14] Stather P., Sidloff D., Rhema I., Choke E., Bown M., Sayers R. (2014). A Review of Current Reporting of Abdominal Aortic Aneurysm Mortality and Prevalence in the Literature. Eur. J. Vasc. Endovasc. Surg..

[15] Sidloff D., Stather P., Dattani N., Bown M., Thompson J., Sayers R., Choke E. (2014). Aneurysm Global Epidemiology Study: Public Health Measures Can Further Reduce Abdominal Aortic Aneurysm Mortality. Circulation.

[16] Bath M.F., Gokani V.J., Sidloff D.A., Jones L.R., Choke E., Sayers R.D., Bown M. (2015). Systematic review of cardiovascular disease and cardiovascular death in patients with a small abdominal aortic aneurysm. BJS.

[17] Hultgren R., Granath F., Swedenborg J. (2007). Different Disease Profiles for Women and Men with Abdominal Aortic Aneurysms. Eur. J. Vasc. Endovasc. Surg..

[18] Ashton H.A., Gao L., Kim L., Druce P.S., Thompson S.G., Scott R.A.P. (2007). Fifteen-year follow-up of a randomized clinical trial of ultrasonographic screening for abdominal aortic aneurysms. Br. J. Surg..

[19] Lederle F.A. (2011). The Rise and Fall of Abdominal Aortic Aneurysm. Circulation.

[20] Sampson U.K., Norman P.E., Fowkes F.G.R., Aboyans V., Song Y., Harrell F.E.H., Forouzanfar M.H., Naghavi M., Denenberg J.O., McDermott M.M. (2014). Estimation of Global and Regional Incidence and Prevalence of Abdominal Aortic Aneurysms 1990 to 2010. Glob. Hear..

[21] Anjum A., von Allmen R., Greenhalgh R., Powell J.T. (2012). Explaining the decrease in mortality from abdominal aortic aneurysm rupture. Br. J. Surg..

[22] Png C.M., Wu J., Tang T.Y., Png I.P., Sheng T.J., Choke E. (2021). Editor’s Choice—Decrease in Mortality from Abdominal Aortic Aneurysms (2001 to 2015): Is it Decreasing Even Faster?. Eur. J. Vasc. Endovasc. Surg..

[23] Karkos C., Mukhopadhyay U., Papakostas I., Ghosh J., Thomson G., Hughes R. (2000). Abdominal Aortic Aneurysm: The Role of Clinical Examination and Opportunistic Detection. Eur. J. Vasc. Endovasc. Surg..

[24] Fink H.A., Lederle F.A., Roth C.S., Bowles C.A., Nelson D.B., Haas M.A. (2000). The Accuracy of Physical Examination to Detect Abdominal Aortic Aneurysm. Arch. Intern. Med..

[25] Schermerhorn M.L., Bensley R.P., Giles K.A., Hurks R., O’Malley A.J., Cotterill P., Chaikof E., Landon B.E. (2012). Changes in Abdominal Aortic Aneurysm Rupture and Short-Term Mortality, 1995–2008: A Retrospective Observational Study. Ann. Surg..

[26] Jeanmonod D., Yelamanchili V.S., Jeanmonod R. (2021). Abdominal Aortic Aneurysm Rupture. StatPearls.

[27] Brady A.R., Thompson S.G., Fowkes F.G.R., Greenhalgh R.M., Powell J.T. (2004). UK Small Aneurysm Trial Participants Abdominal Aortic Aneurysm Expansion: Risk Factors and Time Intervals for Surveillance. Circulation.

[28] Thompson S.G., Brown L.C., Sweeting M., Bown M., Kim L., Glover M., Buxton M.J., Powell J. (2013). Systematic review and meta-analysis of the growth and rupture rates of small abdominal aortic aneurysms: Implications for surveillance intervals and their cost-effectiveness. Heal. Technol. Assess..

[29] Mofidi R., Goldie V.J., Kelman J., Dawson A.R.W., Murie J.A., Chalmers R.T.A. (2007). Influence of sex on expansion rate of abdominal aortic aneurysms. Br. J. Surg..

[30] Sweeting M., Thompson S.G., Brown L.C., Powell J.T. (2012). Meta-analysis of individual patient data to examine factors affecting growth and rupture of small abdominal aortic aneurysms. Br. J. Surg..

[31] Bhak R.H., Lederle F.A., Messina L.M., Wilson S.E., Wininger M., Johnson G.R., Ballard D.J. (2015). Factors Associated With Small Abdominal Aortic Aneurysm Expansion Rate. JAMA Surg..

[32] Oliver-Williams C., Sweeting M., Turton G., Parkin D., Cooper D., Rodd C., Thompson S.G., Earnshaw J.J. (2018). Gloucestershire and Swindon Abdominal Aortic Aneurysm Screening Programme Lessons Learned about Prevalence and Growth Rates of Abdominal Aortic Aneurysms from a 25-Year Ultrasound Population Screening Programme. BJS.

[33] Thompson A.R., Cooper J.A., Ashton H.A., Hafez H. (2010). Growth rates of small abdominal aortic aneurysms correlate with clinical events. BJS.

[34] Wild J., Stather P., Biancari F., Choke E., Earnshaw J., Grant S., Hafez H., Holdsworth R., Juvonen T., Lindholt J. (2013). A Multicentre Observational Study of the Outcomes of Screening Detected Sub-aneurysmal Aortic Dilatation. Eur. J. Vasc. Endovasc. Surg..

[35] Bown M.J., Sweeting M.J., Brown L.C., Powell J.T., Thompson S.G., RESCAN Collaborators (2013). Surveillance Intervals for Small Abdominal Aortic Aneurysms: A Meta-Analysis. JAMA.

[36] Laine M.T., Laukontaus S.J., Kantonen I., Venermo M. (2016). Population-based study of ruptured abdominal aortic aneurysm. BJS.

[37] GBD 2019 Diseases and Injuries Collaborators (2020). Global burden of 369 diseases and injuries in 204 countries and territories, 1990–2019: A systematic analysis for the Global Burden of Disease Study 2019. Lancet.

[38] Sampson U.K., Norman P.E., Fowkes F.G.R., Aboyans V., Song Y., Harrell F.E.H., Forouzanfar M.H., Naghavi M., Denenberg J.O., McDermott M.M. (2014). Global and Regional Burden of Aortic Dissection and Aneurysms: Mortality Trends in 21 World Regions, 1990 to 2010. Glob. Hear..

[39] Fredrik L., Anders W., Kevin M. (2017). Changes in abdominal aortic aneurysm epidemiology. J. Cardiovasc. Surg..

[40] Assar A.N., Zarins C.K. (2009). Ruptured abdominal aortic aneurysm: A surgical emergency with many clinical presentations. Postgrad. Med, J..

[41] Golledge J. (2019). Abdominal aortic aneurysm: Update on pathogenesis and medical treatments. Nat. Rev. Cardiol..

[42] Lee F.-Y., Chen W.-K., Chiu C.-H., Lin C.-L., Kao C.-H., Chen C.-H., Yang T.-Y., Lai C.-Y. (2017). Increased risk of deep vein thrombosis and pulmonary thromboembolism in patients with aortic aneurysms: A nationwide cohort study. PLoS ONE.

[43] Lech C., Swaminathan A. (2017). Abdominal Aortic Emergencies. Emerg. Med. Clin. North Am..

[44] Azhar B., Patel S.R., Holt P.J., Hinchliffe R.J., Thompson M.M., Karthikesalingam A. (2014). Misdiagnosis of Ruptured Abdominal Aortic Aneurysm: Systematic Review and Meta-Analysis. J. Endovasc. Ther..

[45] Schmitz-Rixen T., Keese M., Hakimi M., Peters A., Böckler D., Nelson K., Grundmann R.T. (2016). Ruptured abdominal aortic aneurysm—Epidemiology, predisposing factors, and biology. Langenbeck’s Arch. Surg..

[46] Ulug P., Powell J.T., Sweeting M., Bown M., Thompson S.G., Jones E., Glover M.J. (2016). Meta-analysis of the current prevalence of screen-detected abdominal aortic aneurysm in women. BJS.

[47] Wanhainen A., Themudo R., Ahlström H., Lind L., Johansson L. (2008). Thoracic and abdominal aortic dimension in 70-year-old men and women—A population-based whole-body magnetic resonance imaging (MRI) study. J. Vasc. Surg..

[48] Svensjö S., Björck M., Wanhainen A. (2012). Current prevalence of abdominal aortic aneurysm in 70-year-old women. BJS.

[49] Kent K.C., Zwolak R.M., Egorova N.N., Riles T.S., Manganaro A., Moskowitz A., Gelijns A.C., Greco G. (2010). Analysis of risk factors for abdominal aortic aneurysm in a cohort of more than 3 million individuals. J. Vasc. Surg..

[50] Chabok M., Nicolaides A., Aslam M., Farahmandfar M., Humphries K., Kermani N.Z., Coltart J., Standfield N. (2016). Risk factors associated with increased prevalence of abdominal aortic aneurysm in women. BJS.

[51] Boese A.C., Chang L., Yin K.-J., Chen Y.E., Lee J.-P., Hamblin M.H. (2018). Sex differences in abdominal aortic aneurysms. Am. J. Physiol. Circ. Physiol..

[52] Lederle F.A., Larson J.C., Margolis K.L., Allison M.A., Freiberg M.S., Cochrane B.B., Graettinger W.F., Curb J.D. (2008). Women’s Health Initiative Cohort Study Abdominal Aortic Aneurysm Events in the Women’s Health Initiative: Cohort Study. BMJ.

[53] Norman P., Powell J. (2007). Abdominal Aortic Aneurysm: The Prognosis in Women Is Worse than in Men. Circulation.

[54] McPhee J.T., Hill J.S., Eslami M. (2007). The impact of gender on presentation, therapy, and mortality of abdominal aortic aneurysm in the United States, 2001–2004. J. Vasc. Surg..

[55] Skibba A.A., Evans J.R., Hopkins S.P., Yoon H.R., Katras T., Kalbfleisch J.H., Rush D.S. (2015). Reconsidering gender relative to risk of rupture in the contemporary management of abdominal aortic aneurysms. J. Vasc. Surg..

[56] Ulug P., Sweeting M., von Allmen R., Thompson S.G., Powell J.T., Jones E., Bown M.J., Glover M., Michaels J. (2017). Morphological suitability for endovascular repair, non-intervention rates, and operative mortality in women and men assessed for intact abdominal aortic aneurysm repair: Systematic reviews with meta-analysis. Lancet.

[57] Jahangir E., Lipworth L., Edwards T.L., Kabagambe E.K., Mumma M.T., Mensah G.A., Fazio S., Blot W.J., Sampson U.K.A. (2015). Smoking, sex, risk factors and abdominal aortic aneurysms: A prospective study of 18,782 persons aged above 65 years in the Southern Community Cohort Study. J. Epidemiol. Community Heal..

[58] Jacomelli J., Summers L., Stevenson A., Lees T., Earnshaw J. (2017). Editor’s Choice—Inequalities in Abdominal Aortic Aneurysm Screening in England: Effects of Social Deprivation and Ethnicity. Eur. J. Vasc. Endovasc. Surg..

[59] Perlstein M.D., Gupta S., Ma X., Rong L.Q., Askin G., White R.S. (2019). Abdominal Aortic Aneurysm Repair Readmissions and Disparities of Socioeconomic Status: A Multistate Analysis, 2007–2014. J. Cardiothorac. Vasc. Anesthesia.

[60] Soden P.A., Zettervall S.L., Deery S.E., Hughes K., Stoner M.C., Goodney P.P., Vouyouka A.G., Schermerhorn M.L. (2018). Society for Vascular Surgery Vascular Quality Initiative Black Patients Present with More Severe Vascular Disease and a Greater Burden of Risk Factors than White Patients at Time of Major Vascular Intervention. J. Vasc. Surg..

[61] Deery S.E., O’Donnell T.F., Shean K.E., Darling J.D., Soden P.A., Hughes K., Wang G.J., Schermerhorn M.L. (2018). Society for Vascular Surgery Vascular Quality Initiative Racial Disparities in Outcomes after Intact Abdominal Aortic Aneurysm Repair. J. Vasc. Surg..

[62] Williams T.K., Schneider E.B., Black J.H., Lum Y.W., Freischlag J.A., Perler B.A., Abularrage C.J. (2013). Disparities in Outcomes for Hispanic Patients Undergoing Endovascular and Open Abdominal Aortic Aneurysm Repair. Ann. Vasc. Surg..

[63] Pujades-Rodriguez M., Timmis A., Stogiannis D., Rapsomaniki E., Denaxas S., Shah A.D., Feder G., Kivimäki M., Hemingway H. (2014). Socioeconomic Deprivation and the Incidence of 12 Cardiovascular Diseases in 1.9 Million Women and Men: Implications for Risk Prediction and Prevention. PLoS ONE.

[64] Zommorodi S., Leander K., Roy J., Steuer J., Hultgren R. (2018). Understanding abdominal aortic aneurysm epidemiology: Socioeconomic position affects outcome. J. Epidemiol. Commun. Heal..

[65] Iribarren C., Darbinian J.A., Go A.S., Fireman B.H., Lee C.D., Grey D.P. (2007). Traditional and Novel Risk Factors for Clinically Diagnosed Abdominal Aortic Aneurysm: The Kaiser Multiphasic Health Checkup Cohort Study. Ann. Epidemiol..

[66] Badger S.A., O’Donnell M.E., Sharif M.A., Boyd C.S., Hannon R.J., Lau L.L., Lee B., Soong C.V. (2008). Risk Factors for Abdominal Aortic Aneurysm and the Influence of Social Deprivation. Angiology.

[67] Zarrouk M., Holst J., Malina M., Lindblad B., Wann-Hansson C., Rosvall M., Gottsäter A. (2013). The importance of socioeconomic factors for compliance and outcome at screening for abdominal aortic aneurysm in 65-year-old men. J. Vasc. Surg..

[68] Khashram M., Pitama S., Williman J.A., Jones G.T., Roake J.A. (2017). Survival Disparity Following Abdominal Aortic Aneurysm Repair Highlights Inequality in Ethnic and Socio-economic Status. Eur. J. Vasc. Endovasc. Surg..

[69] Cavelaars A.E.J.M., Kunst A.E., Geurts J.J.M., Crialesi R., Grötvedt L., Helmert U., Lahelma E., Lundberg O., Matheson J., Mielck A. (2000). Educational differences in smoking: International comparison. BMJ.

[70] Wallach-Kildemoes H., Andersen M., Diderichsen F., Lange T. (2013). Adherence to preventive statin therapy according to socioeconomic position. Eur. J. Clin. Pharmacol..

[71] Ultee K., Gonçalves F.B., Hoeks S., Rouwet E., Boersma E., Stolker R., Verhagen H. (2015). Low Socioeconomic Status is an Independent Risk Factor for Survival After Abdominal Aortic Aneurysm Repair and Open Surgery for Peripheral Artery Disease. Eur. J. Vasc. Endovasc. Surg..

[72] Joergensen T., Houlind K., Green A., Lindholt J. (2014). Abdominal Aortic Diameter Is Increased in Males with a Family History of Abdominal Aortic Aneurysms: Results from the Danish VIVA-trial. Eur. J. Vasc. Endovasc. Surg..

[73] Ogata T., MacKean G.L., Cole C.W., Arthur C., Andreou P., Tromp G., Kuivaniemi H. (2005). The lifetime prevalence of abdominal aortic aneurysms among siblings of aneurysm patients is eightfold higher than among siblings of spouses: An analysis of 187 aneurysm families in Nova Scotia, Canada. J. Vasc. Surg..

[74] Larsson E., Granath F., Swedenborg J., Hultgren R. (2009). A population-based case-control study of the familial risk of abdominal aortic aneurysm. J. Vasc. Surg..

[75] Akai A., Watanabe Y., Hoshina K., Obitsu Y., Deguchi J., Sato O., Shigematsu K., Miyata T. (2015). Family history of aortic aneurysm is an independent risk factor for more rapid growth of small abdominal aortic aneurysms in Japan. J. Vasc. Surg..

[76] van de Luijtgaarden K.M., Gonçalves F.B., Hoeks S.E., Majoor-Krakauer D., Rouwet E.V., Stolker R.J., Verhagen H.J. (2014). Familial abdominal aortic aneurysm is associated with more complications after endovascular aneurysm repair. J. Vasc. Surg..

[77] Wanhainen A., Bergqvist D., Boman K., Nilsson T.K., Rutegård J., Björck M. (2005). Risk factors associated with abdominal aortic aneurysm: A population-based study with historical and current data. J. Vasc. Surg..

[78] Sakalihasan N., Defraigne J.-O., Kerstenne M.-A., Cheramy-Bien J.-P., Smelser D.T., Tromp G., Kuivaniemi H. (2014). Family Members of Patients with Abdominal Aortic Aneurysms Are at Increased Risk for Aneurysms: Analysis of 618 Probands and Their Families from the Liège AAA Family Study. Ann. Vasc. Surg..

[79] Kuivaniemi H., Shibamura H., Arthur C., Berguer R., Cole C.W., Juvonen T., Kline R.A., Limet R., MacKean G., Norrgård Ö. (2003). Familial abdominal aortic aneurysms: Collection of 233 multiplex families. J. Vasc. Surg..

[80] Meester J.A.N., Verstraeten A., Schepers D., Alaerts M., Van Laer L., Loeys B.L. (2017). Differences in manifestations of Marfan syndrome, Ehlers-Danlos syndrome, and Loeys-Dietz syndrome. Ann. Cardiothorac. Surg..

[81] Wahlgren C.M., Larsson E., Magnusson P., Hultgren R., Swedenborg J. (2010). Genetic and environmental contributions to abdominal aortic aneurysm development in a twin population. J. Vasc. Surg..

[82] Joergensen T., Christensen K., Lindholt J., Larsen L.A., Green A., Houlind K. (2016). Editor’s Choice—High Heritability of Liability to Abdominal Aortic Aneurysms: A Population Based Twin Study. Eur. J. Vasc. Endovasc. Surg..

[83] Mangum K.D., Farber M.A. (2020). Genetic and epigenetic regulation of abdominal aortic aneurysms. Clin. Genet..

[84] Jones G.T., Tromp G., Kuivaniemi H., Gretarsdottir S., Baas A.F., Giusti B., Strauss E., Hof F.N.V., Webb T., Erdman R. (2017). Meta-Analysis of Genome-Wide Association Studies for Abdominal Aortic Aneurysm Identifies Four New Disease-Specific Risk Loci. Circ. Res..

[85] Bradley D., Badger S., McFarland M., Hughes A. (2016). Abdominal Aortic Aneurysm Genetic Associations: Mostly False? A Systematic Review and Meta-analysis. Eur. J. Vasc. Endovasc. Surg..

[86] Wu Z.-Y., Trenner M., Boon R.A., Spin J.M., Maegdefessel L. (2020). Long noncoding RNAs in key cellular processes involved in aortic aneurysms. Atherosclerosis.

[87] Zalewski D.P., Ruszel K.P., Stępniewski A., Gałkowski D., Bogucki J., Komsta Ł., Kołodziej P., Chmiel P., Zubilewicz T., Feldo M. (2020). Dysregulation of microRNA Modulatory Network in Abdominal Aortic Aneurysm. J. Clin. Med..

[88] Lederle F.A., Johnson G.R., Wilson S.E., Chute E.P., Hye R.J., Makaroun M.S., Barone G.W., Bandyk D., Moneta G.L., Makhoul R.G. (2000). The Aneurysm Detection and Management Study Screening Program: Validation Cohort and Final Results. Aneurysm Detection and Management Veterans Affairs Cooperative Study Investigators. Arch. Intern. Med..

[89] Nyrønning L., Videm V., Romundstad P.R., Hultgren R., Mattsson E. (2019). Female sex hormones and risk of incident abdominal aortic aneurysm in Norwegian women in the HUNT study. J. Vasc. Surg..

[90] Stackelberg O., Björck M., Larsson S., Orsini N., Wolk A. (2014). Sex differences in the association between smoking and abdominal aortic aneurysm. BJS.

[91] Altobelli E., Rapacchietta L., Profeta V.F., Fagnano R. (2018). Risk Factors for Abdominal Aortic Aneurysm in Population-Based Studies: A Systematic Review and Meta-Analysis. Int. J. Environ. Res. Public Health.

[92] Forsdahl S.H., Singh K., Solberg S., Jacobsen B.K. (2009). Risk Factors for Abdominal Aortic Aneurysms: A 7-Year Prospective Study: The Tromsø Study, 1994–2001. Circulation.

[93] Lederle F.A., Nelson D.B., Joseph A.M. (2003). Smokers’ Relative Risk for Aortic Aneurysm Compared with Other Smoking-Related Diseases: A Systematic Review. J. Vasc. Surg..

[94] Pujades-Rodriguez M., George J., Shah A.D., Rapsomaniki E., Denaxas S., West R., Smeeth L., Timmis A., Hemingway H. (2014). Heterogeneous associations between smoking and a wide range of initial presentations of cardiovascular disease in 1 937 360 people in England: Lifetime risks and implications for risk prediction. Int. J. Epidemiol..

[95] Flessenkaemper I., Loddenkemper R., Roll S., Enke-Melzer K., Wurps H., Bauer T. (2015). Screening of COPD patients for abdominal aortic aneurysm. Int. J. Chronic Obstr. Pulm. Dis..

[96] Takagi H., Umemoto T. (2016). A Meta-Analysis of the Association of Chronic Obstructive Pulmonary Disease with Abdominal Aortic Aneurysm Presence. Ann. Vasc. Surg..

[97] Meijer C., Kokje V., van Tongeren R., Hamming J., van Bockel J., Möller G., Lindeman J. (2012). An Association between Chronic Obstructive Pulmonary Disease and Abdominal Aortic Aneurysm beyond Smoking: Results from a Case-Control Study. Eur. J. Vasc. Endovasc. Surg..

[98] Takagi H., Umemoto T. (2016). No association of chronic obstructive pulmonary disease with abdominal aortic aneurysm growth. Hear. Vessel..

[99] Takagi H., Umemoto T. (2017). Association of Chronic Obstructive Pulmonary, Coronary Artery, or Peripheral Artery Disease with Abdominal Aortic Aneurysm Rupture. Int. Angiol..

[100] Siika A., Liljeqvist M.L., Zommorodi S., Nilsson O., Andersson P., Gasser T.C., Roy J., Hultgren R. (2019). A large proportion of patients with small ruptured abdominal aortic aneurysms are women and have chronic obstructive pulmonary disease. PLoS ONE.

[101] Crawford J.D., Chivukula V.K., Haller S., Vatankhah N., Bohannan C.J., Moneta G.L., Rugonyi S., Azarbal A.F. (2016). Aortic outflow occlusion predicts rupture of abdominal aortic aneurysm. J. Vasc. Surg..

[102] Wilmink T.B., Quick C.R., Day N.E. (1999). The association between cigarette smoking and abdominal aortic aneurysms. J. Vasc. Surg..

[103] Aune D., Schlesinger S., Norat T., Riboli E. (2018). Tobacco smoking and the risk of abdominal aortic aneurysm: A systematic review and meta-analysis of prospective studies. Sci. Rep..

[104] Norman P.E., Curci J.A. (2013). Understanding the Effects of Tobacco Smoke on the Pathogenesis of Aortic Aneurysm. Arter. Thromb. Vasc. Biol..

[105] Palazzuoli A., Gallotta M., Guerrieri G., Quatrini I., Franci B., Campagna M.S., Neri E., Benvenuti A., Sassi C., Nuti R. (2008). Prevalence of risk factors, coronary and systemic atherosclerosis in abdominal aortic aneurysm: Comparison with high cardiovascular risk population. Vasc. Heal. Risk Manag..

[106] Toghill B.J., Saratzis A., Bown M.J. (2017). Abdominal aortic aneurysm—An independent disease to atherosclerosis?. Cardiovasc. Pathol..

[107] Elkalioubie A., Haulon S., Duhamel A., Rosa M., Rauch A., Staels B., Susen S., Van Belle E., Dupont A. (2015). Meta-Analysis of Abdominal Aortic Aneurysm in Patients With Coronary Artery Disease. Am. J. Cardiol..

[108] Grøndal N., Søgaard R., Lindholt J. (2015). Baseline prevalence of abdominal aortic aneurysm, peripheral arterial disease and hypertension in men aged 65–74 years from a population screening study (VIVA trial). BJS.

[109] Yao L., Folsom A.R., Alonso A., Lutsey P.L., Pankow J., Guan W., Cheng S., Lederle F.A., Tang W. (2018). Association of carotid atherosclerosis and stiffness with abdominal aortic aneurysm: The atherosclerosis risk in communities (ARIC) study. Atherosclerosis.

[110] Baumgartner I., Hirsch A.T., Abola M.T.B., Cacoub P., Poldermans D., Steg P.G., Creager M.A., Bhatt D.L. (2008). REACH Registry investigators Cardiovascular Risk Profile and Outcome of Patients with Abdominal Aortic Aneurysm in Out-Patients with Atherothrombosis: Data from the Reduction of Atherothrombosis for Continued Health (REACH) Registry. J. Vasc. Surg..

[111] Hernesniemi J.A., Vänni V., Hakala T. (2015). The prevalence of abdominal aortic aneurysm is consistently high among patients with coronary artery disease. J. Vasc. Surg..

[112] Durieux R., Van Damme H., Labropoulos N., Yazici A., Legrand V., Albert A., Defraigne J.-O., Sakalihasan N. (2014). High Prevalence of Abdominal Aortic Aneurysm in Patients with Three-vessel Coronary Artery Disease. Eur. J. Vasc. Endovasc. Surg..

[113] Gianfagna F., Veronesi G., Tozzi M., Tarallo A., Borchini R., Ferrario M.M., Bertù L., Montonati A., Castelli P., Mara L. (2018). RoCAV (Risk of Cardiovascular diseases and abdominal aortic Aneurysm in Varese) Project Investigators Prevalence of Abdominal Aortic Aneurysms in the General Population and in Subgroups at High Cardiovascular Risk in Italy. Results of the RoCAV Population Based Study. Eur. J. Vasc. Endovasc. Surg..

[114] Matthews E.O., Rowbotham S.E., Moxon J.V., Jones R.E., de Ceniga M.V., Golledge J. (2017). Meta-analysis of the association between peripheral artery disease and growth of abdominal aortic aneurysms. BJS.

[115] Takagi H., Umemoto T. (2017). Associations of coronary and peripheral artery disease with presence, expansion, and rupture of abdominal aortic aneurysm—A grin without a cat!. Vasa.

[116] Freiberg M.S., Arnold A.M., Newman A.B., Edwards M.S., Kraemer K.L., Kuller L.H. (2008). Abdominal Aortic Aneurysms, Increasing Infrarenal Aortic Diameter, and Risk of Total Mortality and Incident Cardiovascular Disease Events: 10-Year Follow-up Data from the Cardiovascular Health Study. Circulation.

[117] Goodney P.P., Tavris D., Lucas F.L., Gross T., Fisher E.S., Finlayson S.R.G. (2010). Causes of late mortality after endovascular and open surgical repair of infrarenal abdominal aortic aneurysms. J. Vasc. Surg..

[118] Bahia S.S., Vidal-Diez A., Seshasai S.R.K., Shpitser I., Brownrigg J.R., Patterson B.O., Ray K.K., Holt P.J., Thompson M.M., Karthikesalingam A. (2016). Cardiovascular risk prevention and all-cause mortality in primary care patients with an abdominal aortic aneurysm. BJS.

[119] Kobeissi E., Hibino M., Pan H., Aune D. (2019). Blood pressure, hypertension and the risk of abdominal aortic aneurysms: A systematic review and meta-analysis of cohort studies. Eur. J. Epidemiol..

[120] Takagi H., Umemoto T. (2016). Association of Hypertension with Abdominal Aortic Aneurysm Expansion. Ann. Vasc. Surg..

[121] Takagi H., Manabe H., Kawai N., Goto S.-N., Umemoto T. (2010). Serum High-Density and Low-Density Lipoprotein Cholesterol Is Associated with Abdominal Aortic Aneurysm Presence: A Systematic Review and Meta-Analysis. Int. Angiol..

[122] Stather P.W., Sidloff D.A., Dattani N., Gokani V.J., Choke E., Sayers R.D., Bown M. (2014). Meta-analysis and meta-regression analysis of biomarkers for abdominal aortic aneurysm. BJS.

[123] Rizzo M., Krayenbühl P.-A., Pernice V., Frasheri A., Rini G.B., Berneis K. (2009). LDL size and subclasses in patients with abdominal aortic aneurysm. Int. J. Cardiol..

[124] Takagi H., Umemoto T., Information R. (2016). The association between body mass index and abdominal aortic aneurysm growth: A systematic review. Vasa.

[125] Cronin O., Walker P.J., Golledge J. (2013). The association of obesity with abdominal aortic aneurysm presence and growth. Atherosclerosis.

[126] Apoloni R.C., Zerati A.E., Wolosker N., Saes G.F., Wolosker M., Curado T., Puech-Leão P., De Luccia N. (2019). Analysis of the Correlation Between Central Obesity and Abdominal Aortic Diseases. Ann. Vasc. Surg..

[127] Stackelberg O., Björck M., Azodi O.S., Larsson S., Orsini N., Wolk A. (2013). Obesity and abdominal aortic aneurysm. BJS.

[128] Golledge J., Clancy P., Jamrozik K., Norman P.E. (2007). Obesity, Adipokines, and Abdominal Aortic Aneurysm: Health in Men Study. Circulation.

[129] Cronin O., Liu D., Bradshaw B., Iyer V., Buttner P., Cunningham M., Walker P.J., Golledge J. (2014). Visceral adiposity is not associated with abdominal aortic aneurysm presence and growth. Vasc. Med..

[130] Elagizi A., Kachur S., Lavie C.J., Carbone S., Pandey A., Ortega F.B., Milani R.V. (2018). An Overview and Update on Obesity and the Obesity Paradox in Cardiovascular Diseases. Prog. Cardiovasc. Dis..

[131] Mason R.H., Ruegg G., Perkins J., Hardinge M., Amann-Vesti B., Senn O., Stradling J.R., Kohler M. (2011). Obstructive Sleep Apnea in Patients with Abdominal Aortic Aneurysms: Highly Prevalent and Associated with Aneurysm Expansion. Am. J. Respir. Crit. Care Med..

[132] Bianchi V., Herbert W.G., Myers J., Ribisl P.M., Miller L., Dalman R.L. (2015). Relationship of obstructive sleep apnea and cardiometabolic risk factors in elderly patients with abdominal aortic aneurysm. Sleep Breath..

[133] Kato M., Kubo A., Green F.N., Takagi H. (2019). Meta-analysis of randomized controlled trials on safety and efficacy of exercise training in patients with abdominal aortic aneurysm. J. Vasc. Surg..

[134] Niebauer S., Niebauer J., Dalman R., Myers J. (2021). Effects of Exercise Training on Vascular Markers of Disease Progression in Patients with Small Abdominal Aortic Aneurysms. Am. J. Med..

[135] Aune D., Sen A., Kobeissi E., Hamer M., Norat T., Riboli E. (2020). Physical activity and the risk of abdominal aortic aneurysm: A systematic review and meta-analysis of prospective studies. Sci. Rep..

[136] Stackelberg O., Wolk A., Eliasson K., Hellberg A., Bersztel A., Larsson S.C., Orsini N., Wanhainen A., Björck M. (2017). Lifestyle and Risk of Screening-Detected Abdominal Aortic Aneurysm in Men. J. Am. Hear. Assoc..

[137] Nordkvist S., Sonestedt E., Acosta S. (2018). Adherence to diet recommendations and risk of abdominal aortic aneurysm in the Malmö Diet and Cancer Study. Sci. Rep..

[138] Takagi H., Umemoto T. (2017). Vitamins and Abdominal Aortic Aneurysm. Int. Angiol..

[139] Nsengiyumva V., Krishna S.M., Moran C.S., Moxon J.V., Morton S.K., Clarke M.W., Seto S.-W., Golledge J. (2020). Vitamin D deficiency promotes large rupture-prone abdominal aortic aneurysms and cholecalciferol supplementation limits progression of aneurysms in a mouse model. Clin. Sci..

[140] Pincemail J., Defraigne J.-O., Courtois A., Albert A., Cheramy-Bien J.-P., Sakalihasan N. (2018). Abdominal Aortic Aneurysm (AAA): Is There a Role for the Prevention and Therapy Using Antioxidants?. Curr. Drug Targets.

[141] De Rango P., Farchioni L., Fiorucci B., Lenti M. (2014). Diabetes and Abdominal Aortic Aneurysms. Eur. J. Vasc. Endovasc. Surg..

[142] Xiong J., Wu Z., Chen C., Wei Y., Guo W. (2016). Association between diabetes and prevalence and growth rate of abdominal aortic aneurysms: A meta-analysis. Int. J. Cardiol..

[143] Shantikumar S., Ajjan R., Porter K., Scott D. (2010). Diabetes and the Abdominal Aortic Aneurysm. Eur. J. Vasc. Endovasc. Surg..

[144] Takagi H., Umemoto T. (2016). Diabetes and Abdominal Aortic Aneurysm Growth. Angiology.

[145] Takagi H., Umemoto T. (2016). Negative Association of Diabetes with Rupture of Abdominal Aortic Aneurysm. Diabetes Vasc. Dis. Res..

[146] Canto E.D., Ceriello A., Rydén L., Ferrini M., Hansen T.B., Schnell O., Standl E., Beulens J.W. (2019). Diabetes as a cardiovascular risk factor: An overview of global trends of macro and micro vascular complications. Eur. J. Prev. Cardiol..

[147] Climent E., Benaiges D., Chillarón J.J., Roux J.A.F.-L., Pedro-Botet J. (2018). Diabetes mellitus as a protective factor of abdominal aortic aneurysm: Possible mechanisms. Clin. Investig. Arterioscler..

[148] Miyama N., Dua M.M., Yeung J.J., Schultz G.M., Asagami T., Sho E., Sho M., Dalman R.L. (2010). Hyperglycemia limits experimental aortic aneurysm progression. J. Vasc. Surg..

[149] Dua M.M., Miyama N., Azuma J., Schultz G.M., Sho M., Morser J., Dalman R.L. (2010). Hyperglycemia modulates plasminogen activator inhibitor-1 expression and aortic diameter in experimental aortic aneurysm disease. Surgery.

[150] Thompson A., Cooper J.A., Fabricius M., Humphries S.E., Ashton H.A., Hafez H. (2010). An analysis of drug modulation of abdominal aortic aneurysm growth through 25 years of surveillance. J. Vasc. Surg..

[151] Fujimura N., Xiong J., Kettler E., Xuan H., Glover K.J., Mell M.W., Xu B., Dalman R.L. (2016). Metformin treatment status and abdominal aortic aneurysm disease progression. J. Vasc. Surg..

[152] Golledge J., Moxon J., Pinchbeck J., Anderson G., Rowbotham S., Jenkins J., Bourke M., Bourke B., Dear A., Buckenham T. (2017). Association between metformin prescription and growth rates of abdominal aortic aneurysms. Br. J. Surg..

[153] Gouliamos A.D., Tsiganis T., Dimakakos P., Vlahos L.J. (2004). Screening for abdominal aortic aneurysms during routine lumbar CT scan: Modification of the standard technique. Clin. Imaging.

[154] Argyriou C., Georgiadis G.S., Kontopodis N., Pherwani A.D., Van Herwaarden J.A., Hazenberg C.E., Antoniou G.A. (2018). Screening for Abdominal Aortic Aneurysm During Transthoracic Echocardiography: A Systematic Review and Meta-analysis. Eur. J. Vasc. Endovasc. Surg..

[155] Matsumura Y., Wada M., Hirakawa D., Yasuoka Y., Morimoto N., Takeuchi H., Kitaoka H., Orihashi K., Sugiura T. (2015). Clinical utility of transthoracic echocardiography for screening abdominal aortic aneurysm: A prospective study in a Japanese population. Cardiovasc. Ultrasound.

[156] Aboyans V., Bataille V., Bliscaux P., Ederhy S., Filliol D., Honton B., Kurtz B., Messas E., Mohty D., Brochet E. (2014). Effectiveness of Screening for Abdominal Aortic Aneurysm During Echocardiography. Am. J. Cardiol..

[157] Stather P., Dattani N., Bown M., Earnshaw J., Lees T. (2013). International Variations in AAA Screening. Eur. J. Vasc. Endovasc. Surg..

[158] Wanhainen A., Verzini F., Van Herzeele I., Allaire E., Bown M., Cohnert T., Dick F., van Herwaarden J., Karkos C., Koelemay M. (2019). Editor’s Choice—European Society for Vascular Surgery (ESVS) 2019 Clinical Practice Guidelines on the Management of Abdominal Aorto-iliac Artery Aneurysms. Eur. J. Vasc. Endovasc. Surg..

[159] Thompson S.G., Ashton H.A., Gao L., Buxton M.J., Scott R.A.P. (2012). Multicentre Aneurysm Screening Study (MASS) Group Final Follow-up of the Multicentre Aneurysm Screening Study (MASS) Randomized Trial of Abdominal Aortic Aneurysm Screening. Br. J. Surg..

[160] Lindholt J.S., Sørensen J., Søgaard R., Henneberg E.W. (2010). Long-term benefit and cost-effectiveness analysis of screening for abdominal aortic aneurysms from a randomized controlled trial. BJS.

[161] Cosford P.A., Leng G.C., Thomas J. (2007). Screening for Abdominal Aortic Aneurysm. Cochrane Database Syst. Rev..

[162] Guirguis-Blake J.M., Beil T.L., Senger C.A., Coppola E.L. (2019). Primary Care Screening for Abdominal Aortic Aneurysm: Updated Evidence Report and Systematic Review for the US Preventive Services Task Force. JAMA.

[163] Wanhainen A., Hultgren R., Linné A., Holst J., Gottsäter A., Langenskiöld M., Smidfelt K., Björck M., Svensjö S., Lyttkens L. (2016). Swedish Aneurysm Screening Study Group (SASS) Outcome of the Swedish Nationwide Abdominal Aortic Aneurysm Screening Program. Circulation.

[164] Svensjö S., Mani K., Björck M., Lundkvist J., Wanhainen A. (2014). Screening for Abdominal Aortic Aneurysm in 65-Year-old Men Remains Cost-effective with Contemporary Epidemiology and Management. Eur. J. Vasc. Endovasc. Surg..

[165] Glover M.J., Kim L.G., Sweeting M.J., Thompson S.G., Buxton M.J. (2014). Cost-effectiveness of the National Health Service abdominal aortic aneurysm screening programme in England. BJS.

[166] Bath M.F., Sidloff D., Saratzis A., Bown M.J., Pathak R., Brooks M., Hayes P., Imray C., Quarmby J., Choksy S. (2018). UK Aneurysm Growth Study investigators Impact of Abdominal Aortic Aneurysm Screening on Quality of Life. BJS.

[167] Kapila V., Jetty P., Wooster D., Vucemilo V., Dubois L. (2021). Canadian Society for Vascular Surgery Screening for Abdominal Aortic Aneurysms in Canada: 2020 Review and Position Statement of the Canadian Society for Vascular Surgery. Can. J. Surg..

[168] Ravn H., Wanhainen A., Björck M. (2008). Risk of new aneurysms after surgery for popliteal artery aneurysm. BJS.

[169] Lindholt J., Juul S., Henneberg E. (2007). High-risk and Low-risk Screening for Abdominal Aortic Aneurysm Both Reduce Aneurysm-related Mortality. A Stratified Analysis from a Single-centre Randomised Screening Trial. Eur. J. Vasc. Endovasc. Surg..

[170] Lindholt J., Vammen S., Juul S., Fasting H., Henneberg E.W. (2000). Optimal Interval Screening and Surveillance of Abdominal Aortic Aneurysms. Eur. J. Vasc. Endovasc. Surg..

[171] Greco G., Egorova N.N., Gelijns A.C., Moskowitz A.J., Manganaro A.J., Zwolak R.M., Riles T.S., Kent K.C. (2010). Development of a Novel Scoring Tool for the Identification of Large ≥5 cm Abdominal Aortic Aneurysms. Ann. Surg..

[172] Bobadilla J.L., Kent K.C. (2012). Screening for Abdominal Aortic Aneurysms. Adv. Surg..

[173] Hallett R.L., Ullery B.W., Fleischmann D. (2018). Abdominal aortic aneurysms: Pre- and post-procedural imaging. Abdom. Radiol..

[174] Sever A., Rheinboldt M. (2016). Unstable abdominal aortic aneurysms: A review of MDCT imaging features. Emerg. Radiol..

[175] Kumar Y., Hooda K., Li S., Goyal P., Gupta N., Adeb M. (2017). Abdominal aortic aneurysm: Pictorial review of common appearances and complications. Ann. Transl. Med..

[176] Chaikof E.L., Dalman R.L., Eskandari M.K., Jackson B.M., Lee W.A., Mansour M.A., Mastracci T.M., Mell M., Murad M.H., Nguyen L.L. (2018). The Society for Vascular Surgery practice guidelines on the care of patients with an abdominal aortic aneurysm. J. Vasc. Surg..

[177] Wilmink A., Forshaw M., Quick C., Hubbard C., Day N. (2002). Accuracy of serial screening for abdominal aortic aneurysms by ultrasound. J. Med Screen..

[178] Rubano E., Mehta N., Caputo W., Paladino L., Sinert R. (2013). Systematic Review: Emergency Department Bedside Ultrasonography for Diagnosing Suspected Abdominal Aortic Aneurysm. Acad. Emerg. Med..

[179] Gürtelschmid M., Björck M., Wanhainen A. (2014). Comparison of three ultrasound methods of measuring the diameter of the abdominal aorta. Br. J. Surg..

[180] Grøndal N., Bramsen M., Thomsen M., Rasmussen C., Lindholt J. (2012). The Cardiac Cycle is a Major Contributor to Variability in Size Measurements of Abdominal Aortic Aneurysms by Ultrasound. Eur. J. Vasc. Endovasc. Surg..

[181] Meecham L., Evans R., Buxton P., Allingham K., Hughes M., Rajagopalan S., Fairhead J., Asquith J., Pherwani A. (2015). Abdominal Aortic Aneurysm Diameters: A Study on the Discrepancy between Inner to Inner and Outer to Outer Measurements. Eur. J. Vasc. Endovasc. Surg..

[182] Beales L., Wolstenhulme S., Evans J.A., West R., Scott D.J.A. (2011). Reproducibility of ultrasound measurement of the abdominal aorta. BJS.

[183] Long A., Rouet L., Lindholt J., Allaire E. (2012). Measuring the Maximum Diameter of Native Abdominal Aortic Aneurysms: Review and Critical Analysis. Eur. J. Vasc. Endovasc. Surg..

[184] Brambilla M., Cerini P., Lizio D., Vigna L., Carriero A., Fossaceca R. (2015). Cumulative Radiation Dose and Radiation Risk from Medical Imaging in Patients Subjected to Endovascular Aortic Aneurysm Repair. Radiol. Med..

[185] Ayache J.B., Collins J.D. (2014). MR Angiography of the Abdomen and Pelvis. Radiol. Clin. North Am..

[186] Mitra A., Pencharz D., Davis M., Wagner T. (2018). Determining the Diagnostic Value of 18F-Fluorodeoxyglucose Positron Emission/Computed Tomography in Detecting Prosthetic Aortic Graft Infection. Ann. Vasc. Surg..

[187] Husmann L., Huellner M.W., Ledergerber B., Eberhard N., Kaelin M.B., Anagnostopoulos A., Kudura K., Burger I.A., Mestres C.-A., Rancic Z. (2020). Diagnostic Accuracy of PET/CT and Contrast Enhanced CT in Patients With Suspected Infected Aortic Aneurysms. Eur. J. Vasc. Endovasc. Surg..

[188] Syed M.B., Fletcher A.J., Dweck M.R., Forsythe R., Newby D.E. (2019). Imaging aortic wall inflammation. Trends Cardiovasc. Med..

[189] Brangsch J., Reimann C., Collettini F., Buchert R., Botnar R., Makowski M.R. (2017). Molecular Imaging of Abdominal Aortic Aneurysms. Trends Mol. Med..

[190] Powell J.T., Brown L.C., Forbes J.F., Fowkes F.G.R., Greenhalgh R.M., Ruckley C.V., Thompson S.G. (2007). Final 12-Year Follow-up of Surgery versus Surveillance in the UK Small Aneurysm Trial. Br. J. Surg..

[191] Lederle F.A., Wilson S.E., Johnson G.R., Reinke D.B., Littooy F.N., Acher C.W., Ballard D.J., Messina L.M., Gordon I.L., Chute E.P. (2002). Immediate Repair Compared with Surveillance of Small Abdominal Aortic Aneurysms. N. Engl. J. Med..

[192] Cao P., De Rango P., Verzini F., Parlani G., Romano L., Cieri E. (2011). CAESAR Trial Group Comparison of Surveillance versus Aortic Endografting for Small Aneurysm Repair (CAESAR): Results from a Randomised Trial. Eur. J. Vasc. Endovasc. Surg..

[193] Ouriel K., Clair D.G., Kent K.C., Zarins C.K. (2010). Positive Impact of Endovascular Options for treating Aneurysms Early (PIVOTAL) Investigators Endovascular Repair Compared with Surveillance for Patients with Small Abdominal Aortic Aneurysms. J. Vasc. Surg..

[194] Filardo G., Powell J., Martinez M.A.-M., Ballard D.J. (2015). Surgery for small asymptomatic abdominal aortic aneurysms. Cochrane Database Syst. Rev..

[195] Leemans E., Willems T.P., Van Der Laan M.J., Slump C.H., Zeebregts C.J. (2016). Biomechanical Indices for Rupture Risk Estimation in Abdominal Aortic Aneurysms. J. Endovasc. Ther..

[196] van Walraven C., Wong J., Morant K., Jennings A., Austin P.C., Jetty P., Forster A.J. (2011). The influence of incidental abdominal aortic aneurysm monitoring on patient outcomes. J. Vasc. Surg..

[197] Chun K.C., Schmidt A.S., Bains S., Nguyen A.T., Samadzadeh K.M., Wilson M.D., Peters J.H., Lee E.S. (2016). Surveillance outcomes of small abdominal aortic aneurysms identified from a large screening program. J. Vasc. Surg..

[198] Galiñanes E.L., Reynolds S., Dombrovskiy V.Y., Vogel T.R. (2015). The impact of preoperative statin therapy on open and endovascular abdominal aortic aneurysm repair outcomes. Vascular.

[199] Saratzis A., Dattani N., Brown A., Shalhoub J., Bosanquet D., Sidloff D., Stather P. (2017). Vascular and Endovascular Research Network (VERN) Multi-Centre Study on Cardiovascular Risk Management on Patients Undergoing AAA Surveillance. Eur. J. Vasc. Endovasc. Surg..

[200] Rigotti N.A., Clair C. (2013). Managing tobacco use: The neglected cardiovascular disease risk factor. Eur. Hear. J..

[201] Arinze N., Farber A., Levin S.R., Cheng T.W., Jones D.W., Siracuse C.G., Patel V.I., Rybin D., Doros G., Siracuse J.J. (2019). The effect of the duration of preoperative smoking cessation timing on outcomes after elective open abdominal aortic aneurysm repair and lower extremity bypass. J. Vasc. Surg..

[202] Martínez-González M.A., Gea A., Ruiz-Canela M. (2019). The Mediterranean Diet and Cardiovascular Health. Circ. Res..

[203] Hlebowicz J., Drake I., Gullberg B., Sonestedt E., Wallström P., Persson M., Nilsson J., Hedblad B., Wirfält E. (2013). A High Diet Quality Is Associated with Lower Incidence of Cardiovascular Events in the Malmö Diet and Cancer Cohort. PLoS ONE.

[204] Fiuza-Luces C., Santos-Lozano A., Joyner M., Carrera-Bastos P., Picazo O., Zugaza J.L., Izquierdo M., Ruilope L.M., Lucia A. (2018). Exercise benefits in cardiovascular disease: Beyond attenuation of traditional risk factors. Nat. Rev. Cardiol..

[205] Petridou A., Siopi A., Mougios V. (2019). Exercise in the management of obesity. Metabolism.

[206] Aboyans V., Ricco J.-B., Bartelink M.-L.E.L., Björck M., Brodmann M., Cohnert T., Collet J.-P., Czerny M., De Carlo M., Debus S. (2018). 2017 ESC Guidelines on the Diagnosis and Treatment of Peripheral Arterial Diseases, in collaboration with the European Society for Vascular Surgery (ESVS): Document Covering Atherosclerotic Disease of Extracranial Carotid and Vertebral, Mesenteric, Renal, Upper and Lower Extremity Arteries Endorsed by: The European Stroke Organization (ESO) The Task Force for the Diagnosis and Treatment of Peripheral Arterial Diseases of the European Society of Cardiology (ESC) and of the European Society for Vascular Surgery (ESVS). Eur. Heart J..

[207] Piepoli M.F., Hoes A.W., Agewall S., Albus C., Brotons C., Catapano A.L., Cooney M.T., Corrà U., Cosyns B., Deaton C. (2016). 2016 European Guidelines on Cardiovascular Disease Prevention in Clinical Practice: The Sixth Joint Task Force of the European Society of Cardiology and Other Societies on Cardiovascular Disease Prevention in Clinical Practice (Constituted by Representatives of 10 Societies and by Invited Experts) Developed with the Special Contribution of the European Association for Cardiovascular Prevention & Rehabilitation (EACPR). Atherosclerosis.

[208] Klopf J., Scheuba A., Brostjan C., Neumayer C., Eilenberg W. (2020). Bisherige Strategien und zukünftige Perspektive zur Reduzierung der Wachstumsraten bei abdominellen Aortenaneurysmen: Eine selektive Literaturrecherche sowie Erörterung des aktuellen Vienna MetAAA Trials. Gefässchirurgie.

[209] Yoshimura K., Morikage N., Nishino-Fujimoto S., Furutani A., Shirasawa B., Hamano K. (2018). Current Status and Perspectives on Pharmacologic Therapy for Abdominal Aortic Aneurysm. Curr. Drug Targets.

[210] Kokje V., Hamming J., Lindeman J. (2015). Editor’s Choice—Pharmaceutical Management of Small Abdominal Aortic Aneurysms: A Systematic Review of the Clinical Evidence. Eur. J. Vasc. Endovasc. Surg..

[211] (2002). The Propranolol Aneurysm Trial Investigators Propranolol for small abdominal aortic aneurysms: Results of a randomized trial. J. Vasc. Surg..

[212] Lindholt J.S., Henneberg E.W., Juul S., Fasting H. (1999). Impaired Results of a Randomised Double Blinded Clinical Trial of Propranolol versus Placebo on the Expansion Rate of Small Abdominal Aortic Aneurysms. Int. Angiol..

[213] Wilmink A.B.M., Vardulaki K.A., Hubbard C.S.F., Day N.E., Ashton H.A., Scott A.P., Quick C.R.G. (2002). Are Antihypertensive Drugs Associated with Abdominal Aortic Aneurysms?. J. Vasc. Surg..

[214] Hackam D.G., Thiruchelvam D., Redelmeier D.A. (2006). Angiotensin-converting enzyme inhibitors and aortic rupture: A population-based case-control study. Lancet.

[215] Kortekaas K.E., Meijer C.A., Hinnen J.W., Dalman R.L., Xu B., Hamming J.F., Lindeman J.H. (2014). ACE Inhibitors Potently Reduce Vascular Inflammation, Results of an Open Proof-Of-Concept Study in the Abdominal Aortic Aneurysm. PLoS ONE.

[216] Bicknell C.D., Kiru G., Falaschetti E., Powell J.T., Poulter N.R. (2016). AARDVARK Collaborators An Evaluation of the Effect of an Angiotensin-Converting Enzyme Inhibitor on the Growth Rate of Small Abdominal Aortic Aneurysms: A Randomized Placebo-Controlled Trial (AARDVARK). Eur. Hear. J..

[217] Salata K., Syed M., Hussain M.A., Eikelboom R., de Mestral C., Verma S., Al-Omran M. (2018). Renin-angiotensin system blockade does not attenuate abdominal aortic aneurysm growth, rupture rate, or perioperative mortality after elective repair. J. Vasc. Surg..

[218] Sweeting M., Thompson S.G., Brown L.C., Greenhalgh R.M., Powell J.T. (2010). Use of angiotensin converting enzyme inhibitors is associated with increased growth rate of abdominal aortic aneurysms. J. Vasc. Surg..

[219] Juvonen J., Juvonen T., Laurila A., Alakärppä H., Lounatmaa K., Surcel H.-M., Leinonen M., Kairaluoma M.I., Saikku P. (1997). Demonstration of Chlamydia pneumoniae in the walls of abdominal aortic aneurysms. J. Vasc. Surg..

[220] Halme S., Juvonen T., Laurila A., Mosorin M., Saikku P., Surcel H.-M. (1999). Chlamydia pneumoniaereactive T lymphocytes in the walls of abdominal aortic aneurysms. Eur. J. Clin. Investig..

[221] Baxter B., Pearce W.H., Waltke E.A., Littooy F.N., Hallett J.W., Kent K., Upchurch G.R., Chaikof E.L., Mills J., Fleckten B. (2002). Prolonged administration of doxycycline in patients with small asymptomatic abdominal aortic aneurysms: Report of a prospective (Phase II) multicenter study. J. Vasc. Surg..

[222] Curci J.A., Mao D., Bohner D.G., Allen B.T., Rubin B.G., Reilly J.M., Sicard G.A., Thompson R.W. (2000). Preoperative treatment with doxycycline reduces aortic wall expression and activation of matrix metalloproteinases in patients with abdominal aortic aneurysms. J. Vasc. Surg..

[223] Mosorin M., Juvonen J., Biancari F., Satta J., Surcel H.-M., Leinonen M., Saikku P., Juvonen T. (2001). Use of doxycycline to decrease the growth rate of abdominal aortic aneurysms: A randomized, double-blind, placebo-controlled pilot study. J. Vasc. Surg..

[224] Meijer A., Stijnen T., Wasser M.N., Hamming J.F., Van Bockel J.H., Lindeman J.H. (2013). Pharmaceutical Aneurysm Stabilisation Trial Study Group Doxycycline for Stabilization of Abdominal Aortic Aneurysms: A Randomized Trial. Ann. Intern. Med..

[225] Baxter B.T., Matsumura J., Curci J.A., McBride R., Larson L., Blackwelder W., Lam D., Wijesinha M., Terrin M. (2020). N-TA3CT Investigators Effect of Doxycycline on Aneurysm Growth Among Patients With Small Infrarenal Abdominal Aortic Aneurysms: A Randomized Clinical Trial. JAMA.

[226] Vammen S., Lindholt J., Østergaard L., Fasting H., Henneberg E.W. (2001). Randomized double-blind controlled trial of roxithromycin for prevention of abdominal aortic aneurysm expansion. Br. J. Surg..

[227] Høgh A., Vammen S., Østergaard L., Joensen J.B., Henneberg E.W., Lindholt J. (2009). Intermittent Roxithromycin for Preventing Progression of Small Abdominal Aortic Aneurysms: Long-Term Results of a Small Clinical Trial. Vasc. Endovasc. Surg..

[228] Karlsson L., Gnarpe J., Bergqvist D., Lindbäck J., Pärsson H. (2009). The effect of azithromycin and Chlamydophilia pneumonia infection on expansion of small abdominal aortic aneurysms—A prospective randomized double-blind trial. J. Vasc. Surg..

[229] Takagi H., Yamamoto H., Iwata K., Goto S., Umemoto T. (2012). ALICE (All-Literature Investigation of Cardiovascular Evidence) Group Effects of Statin Therapy on Abdominal Aortic Aneurysm Growth: A Meta-Analysis and Meta-Regression of Observational Comparative Studies. Eur. J. Vasc. Endovasc. Surg..

[230] Salata K., Syed M., Hussain M.A., de Mestral C., Greco E., Mamdani M., Tu J.V., Forbes T.L., Bhatt D.L., Verma S. (2018). Statins Reduce Abdominal Aortic Aneurysm Growth, Rupture, and Perioperative Mortality: A Systematic Review and Meta-Analysis. J. Am. Hear. Assoc..

[231] Pan Z., Cui H., Wu N., Zhang H. (2020). Effect of Statin Therapy on Abdominal Aortic Aneurysm Growth Rate and Mortality: A Systematic Review and Meta-analysis. Ann. Vasc. Surg..

[232] Dunne J.A., Bailey M.A., Griffin K.J., Sohrabi S., Coughlin P.A., Scott D.J.A. (2014). Statins: The Holy Grail of Abdominal Aortic Aneurysm (AAA) Growth Attenuation? A Systematic Review of the Literature. Curr. Vasc. Pharmacol..

[233] Golledge J., Norman P.E., Murphy M.P., Dalman R.L. (2016). Challenges and opportunities in limiting abdominal aortic aneurysm growth. J. Vasc. Surg..

[234] Franklin I.J., Walton L.J., Brown L., Greenhalgh R.N., Powell J.T. (1999). Vascular Surgical Society of Great Britain and Ireland: Non-Steroidal Anti-Inflammatory Drugs to Treat Abdominal Aortic Aneurysm. BJS.

[235] Sillesen H., Eldrup N., Hultgren R., Lindeman J., Bredahl K., Thompson M., Wanhainen A., Wingren U., Swedenborg J., Janson I. (2015). AORTA Trial Investigators Randomized Clinical Trial of Mast Cell Inhibition in Patients with a Medium-Sized Abdominal Aortic Aneurysm. BJS.

[236] Golledge J., Pinchbeck J., Tomee S.M., Rowbotham S.E., Singh T., Moxon J.V., Jenkins J.S., Lindeman J.H., Dalman R.L., McDonnell L. (2020). Efficacy of Telmisartan to Slow Growth of Small Abdominal Aortic Aneurysms: A Randomized Clinical Trial. JAMA Cardiol..

[237] Lindholt J.S., Sorensen H.T., Michel J.B., Thomsen H.F., Henneberg E.W. (2008). Low-Dose Aspirin May Prevent Growth and Later Surgical Repair of Medium-Sized Abdominal Aortic Aneurysms. Vasc. Endovasc. Surg..

[238] Wemmelund H., Jørgensen T.M., Høgh A., Behr-Rasmussen C., Johnsen S.P., Lindholt J.S. (2017). Low-dose aspirin and rupture of abdominal aortic aneurysm. J. Vasc. Surg..

[239] Wanhainen A., Mani K., Kullberg J., Svensjö S., Bersztel A., Karlsson L., Holst J., Gottsäter A., Linné A., Gillgren P. (2020). The Effect of Ticagrelor on Growth of Small Abdominal Aortic Aneurysms-a Randomized Controlled Trial. Cardiovasc. Res..

[240] Clinical Trial Record: Eplerenone in the Management of Abdominal Aortic Aneurysms: A Proof-of-Concept Randomised Controlled Trial. https://clinicaltrials.gov/ct2/show/NCT02345590.

[241] Clinical Trial Record: Brief Administration of Cyclosporine a to Induce the Stabilisation of the Diameter of Small Diameter Abdominal Aortic Aneurysms. https://clinicaltrials.gov/ct2/show/NCT02225756.

[242] Clinical Trial Record: A Prospective Randomized, Double Blind, Placebo-Controlled, Safety and Efficacy Study of Metformin as Add-on Therapy in Non-Diabetic Patients with Abdominal Aortic Aneurysm (MetAAA Study). https://clinicaltrials.gov/ct2/show/NCT03507413.

[243] Clinical Trial Record: Metformin for Abdominal Aortic Aneurysm Growth Inhibition (MAAAGI). https://clinicaltrials.gov/ct2/show/NCT04224051.

[244] Golledge J., Morris D.R., Pinchbeck J., Rowbotham S., Jenkins J., Bourke M., Bourke B., Norman P.E., Jones R., Moxon J.V. (2019). Editor’s Choice—Metformin Prescription is Associated with a Reduction in the Combined Incidence of Surgical Repair and Rupture Related Mortality in Patients with Abdominal Aortic Aneurysm. Eur. J. Vasc. Endovasc. Surg..

[245] Raffort J., Chinetti G., Lareyre F. (2018). Glucagon-Like peptide-1: A new therapeutic target to treat abdominal aortic aneurysm?. Biochimie.

[246] Pini R., Ciavarella C., Faggioli G., Gallitto E., Indelicato G., Fenelli C., Mascoli C., Vacirca A., Gargiulo M., Pasquinelli G. (2020). Different Drugs Effect on Mesenchymal Stem Cells Isolated From Abdominal Aortic Aneurysm. Ann. Vasc. Surg..

[247] Raffort J., Hassen-Khodja R., Jean-Baptiste E., Lareyre F. (2020). Relationship between metformin and abdominal aortic aneurysm. J. Vasc. Surg..

[248] Clinical Trial Record: Limiting AAA With Metformin (LIMIT) Trial (LIMIT). https://clinicaltrials.gov/ct2/show/NCT04500756.

[249] Miyake T., Miyake T., Kurashiki T., Morishita R. (2019). Molecular Pharmacological Approaches for Treating Abdominal Aortic Aneurysm. Ann. Vasc. Dis..

[250] Beck A.W., Sedrakyan A., Mao J., Venermo M., Faizer R., Debus S., Behrendt C.-A., Scali S.T., Altreuther M., Schermerhorn M. (2016). Variations in Abdominal Aortic Aneurysm Care: A Report From the International Consortium of Vascular Registries. Circulation.

[251] Acher C., Ramirez M.C.C., Wynn M. (2020). Operative Mortality and Morbidity in Ruptured Abdominal Aortic Aneurysms in the Endovascular Age. Ann. Vasc. Surg..

[252] D’Oria M., Hanson K.T., Shermerhorn M., Bower T.C., Mendes B.C., Shuja F., Oderich G.S., DeMartino R.R. (2020). Editor’s Choice—Short Term and Long Term Outcomes After Endovascular or Open Repair for Ruptured Infrarenal Abdominal Aortic Aneurysms in the Vascular Quality Initiative. Eur. J. Vasc. Endovasc. Surg..

[253] Ulug P., Powell J.T., Martinez M.A.-M., Ballard D.J., Filardo G. (2020). Surgery for small asymptomatic abdominal aortic aneurysms. Cochrane Database Syst. Rev..

[254] Powell J.T., Wanhainen A. (2020). Analysis of the Differences Between the ESVS 2019 and NICE 2020 Guidelines for Abdominal Aortic Aneurysm. Eur. J. Vasc. Endovasc. Surg..

[255] Lo R.C., Lu B., Fokkema M.T., Conrad M., Patel V.I., Fillinger M., Matyal R., Schermerhorn M.L. (2014). Vascular Study Group of New England, Relative Importance of Aneurysm Diameter and Body Size for Predicting Abdominal Aortic Aneurysm Rupture in Men and Women. J. Vasc. Surg..

[256] Deery S.E., Schermerhorn M.L. (2018). Should Abdominal Aortic Aneurysms in Women be Repaired at a Lower Diameter Threshold?. Vasc. Endovasc. Surg..

[257] Karthaus E.G., Tong T.M.L., Vahl A., Hamming J.F. (2019). Dutch Society of Vascular Surgery, the Steering Committee of the Dutch Surgical Aneurysm Audit and the Dutch Institute for Clinical Auditing Saccular Abdominal Aortic Aneurysms: Patient Characteristics, Clinical Presentation, Treatment, and Outcomes in the Netherlands. Ann. Surg..

[258] Kristmundsson T., Dias N., Resch T., Sonesson B. (2016). Morphology of Small Abdominal Aortic Aneurysms Should be Considered before Continued Ultrasound Surveillance. Ann. Vasc. Surg..

[259] Soden P.A., Zettervall S.L., Ultee K.H., Darling J.D., Buck D.B., Hile C.N., Hamdan A.D., Schermerhorn M.L. (2016). Outcomes for symptomatic abdominal aortic aneurysms in the American College of Surgeons National Surgical Quality Improvement Program. J. Vasc. Surg..

[260] Ten Bosch J.A., Koning S.W., Willigendael E.M., VAN Sambeek M.R., Stokmans R.A., Prins M.H., Teijink J.A. (2016). Symptomatic Abdominal Aortic Aneurysm Repair: To Wait or Not to Wait. J. Cardiovasc. Surg..

[261] Ma B., Wang Y.-N., Chen K., Zhang Y., Pan H., Yang K. (2016). Transperitoneal versus Retroperitoneal Approach for Elective Open Abdominal Aortic Aneurysm Repair. Cochrane Database Syst. Rev..

[262] Wiersema A.M., Jongkind V., Bruijninckx C.M.A., Reijnen M.M.P.J., Vos J.A., van Delden O.M., Zeebregts C.J., Moll F.L. (2012). CAPPAStudy Group Consensus on Arterial PeriProcedural Anticoagulation Prophylactic Perioperative Anti-Thrombotics in Open and Endovascular Abdominal Aortic Aneurysm (AAA) Surgery: A Systematic Review. Eur. J. Vasc. Endovasc. Surg..

[263] Cao P., De Rango P., Parlani G., Verzini F. (2009). Fate of Proximal Aorta Following Open Infrarenal Aneurysm Repair. Semin. Vasc. Surg..

[264] Salata K., Hussain M.A., De Mestral C., Greco E., Aljabri B.A., Mamdani M., Forbes T.L., Bhatt D.L., Verma S., Al-Omran M. (2019). Comparison of Outcomes in Elective Endovascular Aortic Repair vs Open Surgical Repair of Abdominal Aortic Aneurysms. JAMA Netw. Open.

[265] Lee S.Y., Peacock M.R., Farber A., Shah N.K., Eslami M.H., Kalish J.A., Rybin D., Komshian S., Siracuse J.J. (2017). Perioperative Infections after Open Abdominal Aortic Aneurysm Repair Lead to Increased Risk of Subsequent Complications. Ann. Vasc. Surg..

[266] Nicolajsen C.W., Eldrup N. (2020). Abdominal Closure and the Risk of Incisional Hernia in Aneurysm Surgery—A Systematic Review and Meta-analysis. Eur. J. Vasc. Endovasc. Surg..

[267] Indrakusuma R., Jalalzadeh H., van der Meij J.E., Balm R., Koelemay M.J. (2018). Prophylactic Mesh Reinforcement versus Sutured Closure to Prevent Incisional Hernias after Open Abdominal Aortic Aneurysm Repair via Midline Laparotomy: A Systematic Review and Meta-Analysis. Eur. J. Vasc. Endovasc. Surg..

[268] Biancari F., Ylönen K., Anttila V., Juvonen J., Romsi P., Satta J., Juvonen T. (2002). Durability of open repair of infrarenal abdominal aortic aneurysm: A 15-year follow-up study. J. Vasc. Surg..

[269] Conrad M.F., Crawford R.S., Pedraza J.D., Brewster D.C., LaMuraglia G.M., Corey M., Abbara S., Cambria R.P. (2007). Long-term durability of open abdominal aortic aneurysm repair. J. Vasc. Surg..

[270] Paravastu S.C.V., Jayarajasingam R., Cottam R., Palfreyman S.J., Michaels J.A., Thomas S.M. (2014). Endovascular Repair of Abdominal Aortic Aneurysm. Cochrane Database Syst. Rev..

[271] Antoniou G.A., Antoniou S.A. (2020). Editor’s Choice—Percutaneous Access Does Not Confer Superior Clinical Outcomes Over Cutdown Access for Endovascular Aneurysm Repair: Meta-Analysis and Trial Sequential Analysis of Randomised Controlled Trials. Eur. J. Vasc. Endovasc. Surg..

[272] Belvroy V.M., Houben I.B., Trimarchi S., Patel H.J., Moll F.L., Van Herwaarden J.A. (2018). Identifying and addressing the limitations of EVAR technology. Expert Rev. Med. Devices.

[273] Kontopodis N., Galanakis N., Tzartzalou I., Tavlas E., Georgakarakos E., Dimopoulos I., Tsetis D., Ioannou C.V. (2020). An update on the improvement of patient eligibility with the use of new generation endografts for the treatment of abdominal aortic aneurysms. Expert Rev. Med. Devices.

[274] Gopal M., Lau I., Harris J., Berger K., Faries P., Marin M., Tadros R. (2020). A Review of the Evolution of Abdominal Aortic Endografts and Future Directions. Surg. Technol. Int..

[275] Daye D., Walker T.G. (2018). Complications of endovascular aneurysm repair of the thoracic and abdominal aorta: Evaluation and management. Cardiovasc. Diagn. Ther..

[276] Spanos K., Karathanos C., Saleptsis V., Giannoukas A.D. (2015). Systematic review and meta-analysis of migration after endovascular abdominal aortic aneurysm repair. Vascular.

[277] de la Motte L., Falkenberg M., Koelemay M.J., Lönn L. (2018). Is EVAR a Durable Solution? Indications for Reinterventions. J Cardiovasc Surg.

[278] Bradley N.A., Roxburgh C., Khan F., Guthrie G. (2021). Postimplantation syndrome in endovascular aortic aneurysm repair—A systematic review. Vasa.

[279] Holden A. (2018). Aneurysm Repair with Endovascular Aneurysm Sealing: Technique, Patient Selection, and Management of Complications. Tech. Vasc. Interv. Radiol..

[280] Martinelli O., Alunno A., Gattuso R., Di Girolamo A., Irace L. (2021). Nellix endovascular aneurysm-sealing system: A single-center experience and review of current evidence. Futur. Cardiol..

[281] Quaglino S., Mortola L., Ferrero E., Ferri M., Cirillo S., Lario C.V., Negro G., Ricotti A., Gaggiano A. (2020). Long-Term Failure after EVAS in a Real-Life Single Center Experience with the Nellix Endograft. J. Vasc. Surg..

[282] Singh A.A., Benaragama K.S., Pope T., Coughlin P.A., Winterbottom A.P., Harrison S.C., Boyle J.R. (2020). Progressive Device Failure at Long Term Follow Up of the Nellix EndoVascular Aneurysm Sealing (EVAS) System. Eur. J. Vasc. Endovasc. Surg..

[283] Kristensen S.D., Knuuti J., Saraste A., Anker S.D., Bøtker H.E., De Hert S., Ford I., Gonzalez-Juanatey J.R., Gorenek B., Heyndrickx G.R. (2014). 2014 ESC/ESA Guidelines on non-cardiac surgery: Cardiovascular assessment and management: The Joint Task Force on non-cardiac surgery: Cardiovascular assessment and management of the European Society of Cardiology (ESC) and the European Society of Anaesthesiology (ESA). Eur. Hear. J..

[284] Biagi P., de Donato G., Setacci C. (2016). Cardiac Risk Stratification in Patients Undergoing Endovascular Aortic Repair. Minerva Cardioangiol..

[285] Spanos K., Karathanos C., Athanasoulas A., Sapeltsis V., Giannoukas A.D. (2018). Systematic Review of Follow-up Compliance after Endovascular Abdominal Aortic Aneurysm Repair. J. Cardiovasc. Surg (Torino).

[286] Grima M.J., Boufi M., Law M., Jackson D., Stenson K., Patterson B., Loftus I., Thompson M., Karthikesalingam A., Holt P. (2018). Editor’s Choice—The Implications of Non-compliance to Endovascular Aneurysm Repair Surveillance: A Systematic Review and Meta-analysis. Eur. J. Vasc. Endovasc. Surg..

[287] Kim S.H., Litt H.I. (2020). Surveillance Imaging following Endovascular Aneurysm Repair: State of the Art. Semin. Interv. Radiol..

[288] Hu D.K., Pisimisis G.T., Sheth R.A. (2018). Repair of abdominal aortic aneurysms: Preoperative imaging and evaluation. Cardiovasc. Diagn. Ther..

[289] Kudo T. (2019). Surgical Complications after Open Abdominal Aortic Aneurysm Repair: Intestinal Ischemia, Buttock Claudication and Sexual Dysfunction. Ann. Vasc. Dis..

[290] Kouvelos G., Katsargyris A., Antoniou G., Oikonomou K., Verhoeven E. (2016). Outcome after Interruption or Preservation of Internal Iliac Artery Flow During Endovascular Repair of Abdominal Aorto-iliac Aneurysms. Eur. J. Vasc. Endovasc. Surg..

[291] Gaudric J., Tresson P., Derycke L., Du Montcel S.T., Couture T., Davaine J.-M., Kashi M., Lawton J., Chiche L., Koskas F. (2018). Surgical internal iliac artery preservation associated with endovascular repair of infrarenal aortoiliac aneurysms to avoid buttock claudication and distal type I endoleaks. J. Vasc. Surg..

[292] Kalko Y., Ugurlucan M., Basaran M., Aydin U., Kafa U., Kosker T., Suren M., Yasar T. (2007). Epidural anaesthesia and mini-laparotomy for the treatment of abdominal aortic aneurysms in patients with severe chronic obstructive pulmonary disease. Acta Chir. Belg..

[293] Hajibandeh S., Hajibandeh S., Adasonla K., Antoniou S.A., Barrie J., Madan M., Antoniou G.A. (2018). Loco-regional versus general anaesthesia for elective endovascular aneurysm repair—Results of a cohort study and a meta-analysis. Vasa.

[294] Sweeting M.J., Patel R., Powell J.T., Greenhalgh R.M. (2017). EVAR Trial Investigators Endovascular Repair of Abdominal Aortic Aneurysm in Patients Physically Ineligible for Open Repair: Very Long-Term Follow-up in the EVAR-2 Randomized Controlled Trial. Ann. Surg..

[295] Sonesson B., Dias N., Resch T. (2018). Is There an Age Limit for Abdominal Aortic Aneurysm Repair?. J. Cardiovasc. Surg..

[296] Katsargyris A., Lenhardt Michael Florian C., Marques de Marino P., Botos B., Verhoeven E.L. (2020). Reasons for and Outcomes of Open Abdominal Aortic Repair in the Endovascular Era. Ann. Vasc. Surg..

[297] Shan L., Saxena A., Goh D., Robinson D. (2018). A systematic review on the quality of life and functional status after abdominal aortic aneurysm repair in elderly patients with an average age older than 75 years. J. Vasc. Surg..

[298] Nargesi S., Abutorabi A., Alipour V., Tajdini M., Salimi J. (2021). Cost-Effectiveness of Endovascular Versus Open Repair of Abdominal Aortic Aneurysm: A Systematic Review. Cardiovasc. Drugs Ther..

[299] Yokoyama Y., Kuno T., Takagi H. (2020). Meta-analysis of phase-specific survival after elective endovascular versus surgical repair of abdominal aortic aneurysm from randomized controlled trials and propensity score-matched studies. J. Vasc. Surg..

[300] Bahia S., Holt P., Jackson D., Patterson B., Hinchliffe R., Thompson M., Karthikesalingam A. (2015). Systematic Review and Meta-analysis of Long-term survival After Elective Infrarenal Abdominal Aortic Aneurysm Repair 1969–2011: 5 Year Survival Remains Poor Despite Advances in Medical Care and Treatment Strategies. Eur. J. Vasc. Endovasc. Surg..

[301] Khashram M., Williman J., Hider P.N., Jones G.T., Roake J.A. (2017). Management of Modifiable Vascular Risk Factors Improves Late Survival following Abdominal Aortic Aneurysm Repair: A Systematic Review and Meta-Analysis. Ann. Vasc. Surg..

[302] Alberga A.J., Karthaus E.G., van Zwet E.W., de Bruin J.L., van Herwaarden J.A., Wever J.J., Verhagen H.J., Akker P.V.D., Akkersdijk G., Akkersdijk W. (2021). Dutch Institute for Clinical Auditing Outcomes in Octogenarians and the Effect of Comorbidities After Intact Abdominal Aortic Aneurysm Repair in the Netherlands: A Nationwide Cohort Study. Eur. J. Vasc. Endovasc. Surg..

[303] Harris D.G., Bulatao I., Oates C.P., Kalsi R., Drucker C.B., Menon N., Flohr T.R., Crawford R.S. (2017). Functional status predicts major complications and death after endovascular repair of abdominal aortic aneurysms. J. Vasc. Surg..

[304] Al Shakarchi J., Fairhead J., Rajagopalan S., Pherwani A., Jaipersad A. (2020). Impact of Frailty on Outcomes in Patients Undergoing Open Abdominal Aortic Aneurysm Repair. Ann. Vasc. Surg..

[305] Liu Y., Yang Y., Zhao J., Chen X., Wang J., Ma Y., Huang B., Yuan D., Du X. (2020). Systematic review and meta-analysis of sex differences in outcomes after endovascular aneurysm repair for infrarenal abdominal aortic aneurysm. J. Vasc. Surg..

[306] Kalender G., Lisy M., Stock U., Endisch A., Kornberger A. (2019). Long-term radiation exposure in patients undergoing EVAR: Reflecting clinical day-to-day practice to assess realistic radiation burden. Clin. Hemorheol. Microcirc..

[307] Lloyd G., Bown M., Norwood M., Deb R., Fishwick G., Bell P., Sayers R. (2004). Feasibility of preoperative computer tomography in patients with ruptured abdominal aortic aneurysm: A time-to-death study in patients without operation. J. Vasc. Surg..

[308] Biancari F., Paone R., Venermo M., D’Andrea V., Perälä J. (2013). Diagnostic Accuracy of Computed Tomography in Patients with Suspected Abdominal Aortic Aneurysm Rupture. Eur. J. Vasc. Endovasc. Surg..

[309] IMPROVE Trial Investigators (2017). Comparative clinical effectiveness and cost effectiveness of endovascular strategy v open repair for ruptured abdominal aortic aneurysm: Three year results of the IMPROVE randomised trial. BMJ.

[310] Kontopodis N., Galanakis N., Antoniou S.A., Tsetis D., Ioannou C.V., Veith F.J., Powell J.T., Antoniou G.A. (2020). Meta-Analysis and Meta-Regression Analysis of Outcomes of Endovascular and Open Repair for Ruptured Abdominal Aortic Aneurysm. Eur. J. Vasc. Endovasc. Surg..

[311] Varkevisser R.R., Swerdlow N.J., de Guerre L.E., Dansey K., Stangenberg L., Giles K.A., Verhagen H.J., Schermerhorn M.L. (2020). Society for Vascular Surgery Vascular Quality Initiative Five-Year Survival Following Endovascular Repair of Ruptured Abdominal Aortic Aneurysms Is Improving. J. Vasc. Surg..

[312] Li Y., Li Z., Wang S., Chang G., Wu R., Hu Z., Yin H., Wang J., Yao C. (2016). Endovascular versus Open Surgery Repair of Ruptured Abdominal Aortic Aneurysms in Hemodynamically Unstable Patients: Literature Review and Meta-Analysis. Ann. Vasc. Surg..

[313] Kontopodis N., Tavlas E., Ioannou C.V., Giannoukas A.D., Geroulakos G., Antoniou G.A. (2020). Systematic Review and Meta-Analysis of Outcomes of Open and Endovascular Repair of Ruptured Abdominal Aortic Aneurysm in Patients with Hostile vs. Friendly Aortic Anatomy. Eur. J. Vasc. Endovasc. Surg..

[314] Morbiducci U., Kok A.M., Kwak B., Stone P.H., Steinman D.A., Wentzel J.J. (2016). Atherosclerosis at arterial bifurcations: Evidence for the role of haemodynamics and geometry. Thromb. Haemost..

[315] Chistiakov D.A., Orekhov A.N., Bobryshev Y.V. (2016). Effects of shear stress on endothelial cells: Go with the flow. Acta Physiol..

[316] Golledge J., Norman P.E. (2010). Atherosclerosis and Abdominal Aortic Aneurysm: Cause, Response, or Common Risk Factors?. Arter. Thromb. Vasc. Biol..

[317] Johnsen S.H., Forsdahl S.H., Singh K., Jacobsen B.K. (2010). Atherosclerosis in Abdominal Aortic Aneurysms: A Causal Event or a Process Running in Parallel? The Tromsø Study. Arter. Thromb. Vasc. Biol..

[318] Wang D.H., Makaroun M.S., Webster M.W., Vorp D. (2002). Effect of intraluminal thrombus on wall stress in patient-specific models of abdominal aortic aneurysm. J. Vasc. Surg..

[319] Georgakarakos E., Ioannou C.V., Volanis S., Papaharilaou Y., Ekaterinaris J., Katsamouris A.N. (2009). The Influence of Intraluminal Thrombus on Abdominal Aortic Aneurysm Wall Stress. Int. Angiol..

[320] Gasser T.C., Görgülü G., Folkesson M., Swedenborg J. (2008). Failure properties of intraluminal thrombus in abdominal aortic aneurysm under static and pulsating mechanical loads. J. Vasc. Surg..

[321] Roy J., Labruto F., Beckman M.O., Danielson J., Johansson G., Swedenborg J. (2008). Bleeding into the intraluminal thrombus in abdominal aortic aneurysms is associated with rupture. J. Vasc. Surg..

[322] Talvitie M., Liljeqvist M.L., Siika A., Hultgren R., Roy J. (2018). Localized Hyperattenuations in the Intraluminal Thrombus May Predict Rupture of Abdominal Aortic Aneurysms. J. Vasc. Interv. Radiol..

[323] Haller S.J., Crawford J.D., Courchaine K.M., Bohannan C.J., Landry G.J., Moneta G.L., Azarbal A.F., Rugonyi S. (2018). Intraluminal thrombus is associated with early rupture of abdominal aortic aneurysm. J. Vasc. Surg..

[324] Wiernicki I., Parafiniuk M., Kolasa-Wołosiuk A., Gutowska I., Kazimierczak A., Clark J., Baranowska-Bosiacka I., Szumilowicz P., Gutowski P. (2019). Relationship between aortic wall oxidative stress/proteolytic enzyme expression and intraluminal thrombus thickness indicates a novel pathomechanism in the progression of human abdominal aortic aneurysm. FASEB J..

[325] Tong J., Holzapfel G.A. (2015). Structure, Mechanics, and Histology of Intraluminal Thrombi in Abdominal Aortic Aneurysms. Ann. Biomed. Eng..

[326] Koole D., Zandvoort H.J., Schoneveld A., Vink A., Vos J.A., Hoogen L.L.V.D., de Vries J.-P.P., Pasterkamp G., Moll F.L., van Herwaarden J.A. (2013). Intraluminal abdominal aortic aneurysm thrombus is associated with disruption of wall integrity. J. Vasc. Surg..

[327] Khan J.A., Rahman M.A., Mazari F., Shahin Y., Smith G., Madden L., Fagan M., Greenman J., McCollum P., Chetter I. (2012). Intraluminal Thrombus has a Selective Influence on Matrix Metalloproteinases and Their Inhibitors (Tissue Inhibitors of Matrix Metalloproteinases) in the Wall of Abdominal Aortic Aneurysms. Ann. Vasc. Surg..

[328] Michel J.-B., Martin-Ventura J.-L., Egido J., Sakalihasan N., Treska V., Lindholt J., Allaire E., Thorsteinsdottir U., Cockerill G., Swedenborg J. (2011). Novel aspects of the pathogenesis of aneurysms of the abdominal aorta in humans. Cardiovasc. Res..

[329] Zhu C., Leach J.R., Wang Y., Gasper W., Saloner D., Hope M.D. (2020). Intraluminal Thrombus Predicts Rapid Growth of Abdominal Aortic Aneurysms. Radiology.

[330] Nguyen V., Leiner T., Hellenthal F., Backes W., Wishaupt M., van der Geest R., Heeneman S., Kooi M., Schurink G. (2014). Abdominal Aortic Aneurysms with High Thrombus Signal Intensity on Magnetic Resonance Imaging are Associated with High Growth Rate. Eur. J. Vasc. Endovasc. Surg..

[331] The MA^3^RS Study Investigators (2017). Aortic Wall Inflammation Predicts Abdominal Aortic Aneurysm Expansion, Rupture, and Need for Surgical Repair. Circulation.

[332] Behr-Rasmussen C., Grøndal N., Bramsen M., Thomsen M., Lindholt J. (2014). Mural Thrombus and the Progression of Abdominal Aortic Aneurysms: A Large Population-based Prospective Cohort Study. Eur. J. Vasc. Endovasc. Surg..

[333] Parr A., McCann M., Bradshaw B., Shahzad A., Buttner P., Golledge J. (2011). Thrombus volume is associated with cardiovascular events and aneurysm growth in patients who have abdominal aortic aneurysms. J. Vasc. Surg..

[334] Golledge J., Iyer V., Jenkins J., Bradshaw B., Cronin O., Walker P.J. (2013). Thrombus volume is similar in patients with ruptured and intact abdominal aortic aneurysms. J. Vasc. Surg..

[335] Folkesson M., Silveira A., Eriksson P., Swedenborg J. (2011). Protease activity in the multi-layered intra-luminal thrombus of abdominal aortic aneurysms. Atherosclerosis.

[336] Ramos-Mozo P., Madrigal-Matute J., de Ceniga M.V., Blanco-Colio L.M., Meilhac O., Feldman L., Michel J.-B., Clancy P., Golledge J., Norman P.E. (2012). Increased plasma levels of NGAL, a marker of neutrophil activation, in patients with abdominal aortic aneurysm. Atherosclerosis.

[337] Houard X., Touat Z., Ollivier V., Louedec L., Philippe M., Sebbag U., Meilhac O., Rossignol P., Michel J.-B. (2009). Mediators of neutrophil recruitment in human abdominal aortic aneurysms. Cardiovasc. Res..

[338] Zhu C., Silveira A., Hemdahl A.-L., Hamsten A., Hedin U., Swedenborg J., Folkesson M., Kazil M., Eriksson P. (2007). Presence of NGAL/MMP-9 complexes in human abdominal aortic aneurysms. Thromb. Haemost..

[339] Piechota-Polanczyk A., Ejózkowicz A., Nowak W., Eilenberg W., Eneumayer C., Emalinski T., Ehuk I., Ebrostjan C. (2015). The Abdominal Aortic Aneurysm and Intraluminal Thrombus: Current Concepts of Development and Treatment. Front. Cardiovasc. Med..

[340] Sánchez-Infantes D., Nus M., Navas-Madroñal M., Fité J., Pérez B., Barros-Membrilla A., Soto B., Martínez-González J., Camacho M., Rodriguez C. (2021). Oxidative Stress and Inflammatory Markers in Abdominal Aortic Aneurysm. Antioxidants.

[341] Yuan Z., Lu Y., Wei J., Wu J., Yang J., Cai Z. (2020). Abdominal Aortic Aneurysm: Roles of Inflammatory Cells. Front. Immunol.

[342] Cafueri G., Parodi F., Pistorio A., Bertolotto M.B., Ventura F., Gambini C., Bianco P., Dallegri F., Pistoia V., Pezzolo A. (2012). Endothelial and Smooth Muscle Cells from Abdominal Aortic Aneurysm Have Increased Oxidative Stress and Telomere Attrition. PLoS ONE.

[343] Emeto T., Moxon J., Au M., Golledge J. (2016). Oxidative stress and abdominal aortic aneurysm: Potential treatment targets. Clin. Sci..

[344] Morgan S., Yamanouchi D., Harberg C., Wang Q., Keller M., Si Y., Burlingham W., Seedial S., Lengfeld J., Liu B. (2012). Elevated Protein Kinase C-δ Contributes to Aneurysm Pathogenesis Through Stimulation of Apoptosis and Inflammatory Signaling. Arter. Thromb. Vasc. Biol..

[345] Rowe V.L., Stevens S.L., Reddick T.T., Freeman M.B., Donnell R., Carroll R.C., Goldman M.H. (2000). Vascular Smooth Muscle Cell Apoptosis in Aneurysmal, Occlusive, and Normal Human Aortas. J. Vasc. Surg..

[346] Zhang J., Schmidt J., Ryschich E., Schumacher H., Allenberg J.R. (2003). Increased Apoptosis and Decreased Density of Medial Smooth Muscle Cells in Human Abdominal Aortic Aneurysms. Chin. Med. J..

[347] Choke E., Thompson M.M., Dawson J., Wilson W.R.W., Sayed S., Loftus I.M., Cockerill G.W. (2006). Abdominal Aortic Aneurysm Rupture Is Associated With Increased Medial Neovascularization and Overexpression of Proangiogenic Cytokines. Arter. Thromb. Vasc. Biol..

[348] Choke E., Cockerill G.W., Dawson J., Wilson R.W., Jones A., Loftus I.M., Thompson M.M. (2006). Increased Angiogenesis at the Site of Abdominal Aortic Aneurysm Rupture. Ann. New York Acad. Sci..

[349] Blassova T., Tonar Z., Tomasek P., Hosek P., Hollan I., Treska V., Molacek J. (2019). Inflammatory cell infiltrates, hypoxia, vascularization, pentraxin 3 and osteoprotegerin in abdominal aortic aneurysms—A quantitative histological study. PLoS ONE.

[350] Mäyränpää M.I., Trosien J.A., Fontaine V., Folkesson M., Kazi M., Eriksson P., Swedenborg J., Hedin U. (2009). Mast cells associate with neovessels in the media and adventitia of abdominal aortic aneurysms. J. Vasc. Surg..

[351] Ramos-Mozo P., Madrigal-Matute J., Martinez-Pinna R., Blanco-Colio L.M., Lopez J.A., Camafeita E., Meilhac O., Michel J.-B., Aparicio C., de Ceniga M.V. (2011). Proteomic Analysis of Polymorphonuclear Neutrophils Identifies Catalase as a Novel Biomarker of Abdominal Aortic Aneurysm: Potential Implication of Oxidative Stress in Abdominal Aortic Aneurysm Progression. Arter. Thromb. Vasc. Biol..

[352] Lucas M.L., Carraro C.C., Belló-Klein A., Kalil A.N., Aerts N.R., Carvalho F.B., Fernandes M.C., Zettler C.G. (2018). Oxidative Stress in Aortas of Patients with Advanced Occlusive and Aneurysmal Diseases. Ann. Vasc. Surg..

[353] Guzik B., Sagan A., Ludew D., Mrowiecki W., Chwała M., Bujak-Gizycka B., Filip G., Grudzien G., Kapelak B., Żmudka K. (2013). Mechanisms of oxidative stress in human aortic aneurysms—Association with clinical risk factors for atherosclerosis and disease severity. Int. J. Cardiol..

[354] Zhang J., Schmidt J., Ryschich E., Mueller-Schilling M., Schumacher H., Allenberg J.R. (2003). Inducible nitric oxide synthase is present in human abdominal aortic aneurysm and promotes oxidative vascular injury. J. Vasc. Surg..

[355] Ho Y., Wu M.-L., Gung P.-Y., Chen C.-H., Kuo C.-C., Yet S.-F. (2016). Heme oxygenase-1 deficiency exacerbates angiotensin II-induced aortic aneurysm in mice. Oncotarget.

[356] Parastatidis I., Weiss D., Joseph G., Taylor W.R. (2013). Overexpression of Catalase in Vascular Smooth Muscle Cells Prevents the Formation of Abdominal Aortic Aneurysms. Arter. Thromb. Vasc. Biol..

[357] Yu Z., Morimoto K., Yu J., Bao W., Okita Y., Okada K. (2015). Endogenous superoxide dismutase activation by oral administration of riboflavin reduces abdominal aortic aneurysm formation in rats. J. Vasc. Surg..

[358] Xiong W., Mactaggart J., Knispel R., Worth J., Zhu Z., Li Y., Sun Y., Baxter B.T., Johanning J. (2009). Inhibition of reactive oxygen species attenuates aneurysm formation in a murine model. Atherosclerosis.

[359] Jones B., Tonniges J.R., Debski A., Albert B., Yeung D.A., Gadde N., Mahajan A., Sharma N., Calomeni E.P., Go M.R. (2020). Collagen fibril abnormalities in human and mice abdominal aortic aneurysm. Acta Biomater..

[360] Niestrawska J.A., Regitnig P., Viertler C., Cohnert T.U., Babu A.R., Holzapfel G.A. (2019). The role of tissue remodeling in mechanics and pathogenesis of abdominal aortic aneurysms. Acta Biomater..

[361] Krishna S.M., Seto S.W., Jose R., Li J., Moxon J., Clancy P., Crossman D.J., Norman P., Emeto T.I., Golledge J. (2017). High serum thrombospondin-1 concentration is associated with slower abdominal aortic aneurysm growth and deficiency of thrombospondin-1 promotes angiotensin II induced aortic aneurysm in mice. Clin. Sci..

[362] Mordi I.R., Forsythe R.O., Gellatly C., Iskandar Z., McBride O.M., Saratzis A., Chalmers R., Chin C., Bown M.J., Newby D.E. (2019). Plasma Desmosine and Abdominal Aortic Aneurysm Disease. J. Am. Hear. Assoc..

[363] Holsti M., Wanhainen A., Lundin C., Björck M., Tegler G., Svensson J., Sund M. (2018). Circulating Vascular Basement Membrane Fragments are Associated with the Diameter of the Abdominal Aorta and Their Expression Pattern is Altered in AAA Tissue. Eur. J. Vasc. Endovasc. Surg..

[364] Williams H., Johnson J.L., Jackson C.L., White S.J., George S.J. (2010). MMP-7 mediates cleavage of N-cadherin and promotes smooth muscle cell apoptosis. Cardiovasc. Res..

[365] Lyon C.A., Williams H., Bianco R., Simmonds S., Brown B.A., Wadey K.S., Smith F.C.T., Johnson J.L., George S.J. (2017). Aneurysm Severity is Increased by Combined Mmp-7 Deletion and N-cadherin Mimetic (EC4-Fc) Over-Expression. Sci. Rep..

[366] Geraghty P., Rogan M.P., Greene C.M., Boxio R.M.M., Poiriert T., O’Mahony M., Belaaouaj A., O’Neill S.J., Taggart C.C., McElvaney N.G. (2007). Neutrophil elastase up-regulates cathepsin B and matrix metalloprotease-2 expression. J. Immunol..

[367] Tchougounova E., Lundequist A., Fajardo I., Winberg J.-O., Åbrink M., Pejler G. (2005). A Key Role for Mast Cell Chymase in the Activation of Pro-matrix Metalloprotease-9 and Pro-matrix Metalloprotease-2. J. Biol. Chem..

[368] Krotova K., Khodayari N., Oshins R., Aslanidi G., Brantly M.L. (2020). Neutrophil elastase promotes macrophage cell adhesion and cytokine production through the integrin-Src kinases pathway. Sci. Rep..

[369] Chamberlain C.M., Ang L.S., Boivin W.A., Cooper D.M., Williams S.J., Zhao H., Hendel A., Folkesson M., Swedenborg J., Allard M.F. (2010). Perforin-Independent Extracellular Granzyme B Activity Contributes to Abdominal Aortic Aneurysm. Am. J. Pathol..

[370] Erdozain O.J., Pegrum S., Winrow V.R., Horrocks M., Stevens C.R. (2011). Hypoxia in Abdominal Aortic Aneurysm Supports a Role for HIF-1α and Ets-1 as Drivers of Matrix Metalloproteinase Upregulation in Human Aortic Smooth Muscle Cells. J. Vasc. Res..

[371] Tsai S.-H., Huang P.-H., Hsu Y.-J., Peng Y.-J., Lee C.-H., Wang J.-C., Chen J.-W., Lin S.-J. (2016). Inhibition of hypoxia inducible factor-1α attenuates abdominal aortic aneurysm progression through the down-regulation of matrix metalloproteinases. Sci. Rep..

[372] Rabkin S.W. (2017). The Role Matrix Metalloproteinases in the Production of Aortic Aneurysm. Progress Mol. Biol. Transl. Sci..

[373] Kurianiuk A., Socha K., Gacko M., Blachnio-Zabielska A., Karwowska A. (2018). The Relationship between the Concentration of Cathepsin A, D, and E and the Concentration of Copper and Zinc, and the Size of the Aneurysmal Enlargement in the Wall of the Abdominal Aortic Aneurysm. Ann. Vasc. Surg..

[374] Lv B.-J., Lindholt J.S., Wang J., Cheng X., Shi G.-P. (2013). Plasma levels of cathepsins L, K, and V and risks of abdominal aortic aneurysms: A randomized population-based study. Atherosclerosis.

[375] Lv B.-J., Lindholt J., Cheng X., Wang J., Shi G.-P. (2012). Plasma Cathepsin S and Cystatin C Levels and Risk of Abdominal Aortic Aneurysm: A Randomized Population–Based Study. PLoS ONE.

[376] Lorelli D.R., Jean-Claude J.M., Fox C.J., Clyne J., Cambria R.A., Seabrook G.R., Towne J.B. (2002). Response of plasma matrix metalloproteinase-9 to conventional abdominal aortic aneurysm repair or endovascular exclusion: Implications for endoleak. J. Vasc. Surg..

[377] Watanabe T., Sato A., Sawai T., Uzuki M., Goto H., Yamashita H., Akamatsu D., Sato H., Shimizu T., Miyama N. (2006). The Elevated Level of Circulating Matrix Metalloproteinase-9 in Patients with Abdominal Aortic Aneurysms Decreased to Levels Equal to Those of Healthy Controls after an Aortic Repair. Ann. Vasc. Surg..

[378] Qin Y., Cao X., Guo J., Zhang Y., Pan L., Zhang H., Li H., Tang C., Du J., Shi G.-P. (2012). Deficiency of cathepsin S attenuates angiotensin II-induced abdominal aortic aneurysm formation in apolipoprotein E-deficient mice. Cardiovasc. Res..

[379] Sun J., Sukhova G.K., Zhang J., Chen H., Sjöberg S., Libby P., Xia M., Xiong N., Gelb B.D., Shi G.-P. (2012). Cathepsin K Deficiency Reduces Elastase Perfusion–Induced Abdominal Aortic Aneurysms in Mice. Arter. Thromb. Vasc. Biol..

[380] Sun J., Sukhova G.K., Zhang J., Chen H., Sjöberg S., Libby P., Xiang M., Wang J., Peters C., Reinheckel T. (2011). Cathepsin L Activity Is Essential to Elastase Perfusion–Induced Abdominal Aortic Aneurysms in Mice. Arter. Thromb. Vasc. Biol..

[381] Pyo R., Lee J.K., Shipley J.M., Curci J.A., Mao D., Ziporin S.J., Ennis T.L., Shapiro S.D., Senior R.M., Thompson R.W. (2000). Targeted gene disruption of matrix metalloproteinase-9 (gelatinase B) suppresses development of experimental abdominal aortic aneurysms. J. Clin. Investig..

[382] Longo G.M., Xiong W., Greiner T.C., Zhao Y., Fiotti N., Baxter B.T. (2002). Matrix metalloproteinases 2 and 9 work in concert to produce aortic aneurysms. J. Clin. Investig..

[383] Pagano M.B., Zhou H.-F., Ennis T.L., Wu X., Lambris J., Atkinson J.P., Thompson R.W., Hourcade D.E., Pham C.T. (2009). Complement-Dependent Neutrophil Recruitment Is Critical for the Development of Elastase-Induced Abdominal Aortic Aneurysm. Circulation.

[384] Martinez-Pinna R., Madrigal-Matute J., Tarin C., Burillo E., Esteban-Salan M., Pastor-Vargas C., Lindholt J.S., Lopez J.A., Calvo E., de Ceniga M.V. (2013). Proteomic Analysis of Intraluminal Thrombus Highlights Complement Activation in Human Abdominal Aortic Aneurysms. Arter. Thromb. Vasc. Biol..

[385] Martin-Ventura J.L., Martinez-Lopez D., Roldan-Montero R., Gomez-Guerrero C., Blanco-Colio L.M. (2019). Role of complement system in pathological remodeling of the vascular wall. Mol. Immunol..

[386] Bobryshev Y.Y.V., Lord R.S. (2001). Vascular-Associated Lymphoid Tissue (VALT) Involvement in Aortic Aneurysm. Atherosclerosis.

[387] Spear R., Boytard L., Blervaque R., Chwastyniak M., Hot D., Vanhoutte J., Staels B., Lemoine Y., Lamblin N., Pruvot F.-R. (2015). Adventitial Tertiary Lymphoid Organs as Potential Source of MicroRNA Biomarkers for Abdominal Aortic Aneurysm. Int. J. Mol. Sci..

[388] Kugo H., Moriyama T., Zaima N. (2018). Adipocytes and Abdominal Aortic Aneurysm: Putative Potential Role of Adipocytes in the Process of AAA Development. Curr. Drug Targets.

[389] Folkesson M., Vorkapic E., Gulbins E., Japtok L., Kleuser B., Welander M., Länne T., Wågsäter D. (2017). Inflammatory cells, ceramides, and expression of proteases in perivascular adipose tissue adjacent to human abdominal aortic aneurysms. J. Vasc. Surg..

[390] Piacentini L., Werba J.P., Bono E., Saccu C., Tremoli E., Spirito R., Colombo G. (2019). Genome-Wide Expression Profiling Unveils Autoimmune Response Signatures in the Perivascular Adipose Tissue of Abdominal Aortic Aneurysm. Arter. Thromb. Vasc. Biol..

[391] Teo F.H., de Oliveira R.T.D., Villarejos L., Mamoni R.L., Altemani A., Menezes F.H., Blotta M.H.S.L. (2018). Characterization of CD4+ T Cell Subsets in Patients with Abdominal Aortic Aneurysms. Mediat. Inflamm..

[392] Eagleton M.J. (2012). Inflammation in abdominal aortic aneurysms: Cellular infiltrate and cytokine profiles. Vascular.

[393] Middleton R.K., Lloyd G.M., Bown M., Cooper N.J., London N.J., Sayers R.D. (2007). The pro-inflammatory and chemotactic cytokine microenvironment of the abdominal aortic aneurysm wall: A protein array study. J. Vasc. Surg..

[394] Zhou H.-F., Yan H., Cannon J.L., Springer L.E., Green J.M., Pham C.T.N. (2013). CD43-mediated IFN-γ production by CD8+ T cells promotes abdominal aortic aneurysm in mice. J. Immunol..

[395] Sharma A.K., Lu G., Jester A., Johnston W., Zhao Y., Hajzus V.A., Saadatzadeh M.R., Su G., Bhamidipati C.M., Mehta G.S. (2012). Experimental Abdominal Aortic Aneurysm Formation Is Mediated by IL-17 and Attenuated by Mesenchymal Stem Cell Treatment. Circulation.

[396] Wei Z., Wang Y., Zhang K., Liao Y., Ye P., Wu J., Wang Y., Li F., Yao Y., Zhou Y. (2014). Inhibiting the Th17/IL-17A–Related Inflammatory Responses with Digoxin Confers Protection Against Experimental Abdominal Aortic Aneurysm. Arter. Thromb. Vasc. Biol..

[397] Prucha M., Sedivy P., Stadler P., Zdrahal P., Prokopova P., Voska L., Sedlackova L. (2019). Abdominal aortic aneurysm as an IgG4-related disease. Clin. Exp. Immunol..

[398] Li J., Deng Z., Zhang X., Liu F., Yang C., Shi G. (2020). Deficiency of immunoglobulin E protects mice from experimental abdominal aortic aneurysms. FASEB J..

[399] Ait-Oufella H., Wang Y., Herbin O., Bourcier S., Potteaux S., Joffre J., Loyer X., Ponnuswamy P., Esposito B., Dalloz M. (2013). Natural Regulatory T Cells Limit Angiotensin II–Induced Aneurysm Formation and Rupture in Mice. Arter. Thromb. Vasc. Biol..

[400] Zhou Y., Wu W., Lindholt J.S., Sukhova G.K., Libby P., Yu X., Shi G.-P. (2015). Regulatory T cells in human and angiotensin II-induced mouse abdominal aortic aneurysms. Cardiovasc. Res..

[401] Zhu H., Qu X., Zhang C., Yu Y. (2019). Interleukin-10 Promotes Proliferation of Vascular Smooth Muscle Cells by Inhibiting Inflammation in Rabbit Abdominal Aortic Aneurysm. Int. J. Clin. Exp. Pathol..

[402] Meng X., Yang J., Dong M., Zhang K., Tu E., Gao Q., Chen W., Zhang C., Zhang Y. (2015). Regulatory T cells in cardiovascular diseases. Nat. Rev. Cardiol..

[403] Eliason J.L., Hannawa K.K., Ailawadi G., Sinha I., Ford J.W., Deogracias M.P., Roelofs K.J., Woodrum D.T., Ennis T.L., Henke P.K. (2005). Neutrophil Depletion Inhibits Experimental Abdominal Aortic Aneurysm Formation. Circulation.

[404] Daugherty A., Rateri D.L., Charo I.F., Owens A.P., Howatt D.A., Cassis L.A. (2010). Angiotensin II infusion promotes ascending aortic aneurysms: Attenuation by CCR2 deficiency in apoE−/− mice. Clin. Sci..

[405] Xiong W., Knispel R., MacTaggart J., Greiner T.C., Weiss S.J., Baxter B.T. (2009). Membrane-type 1 Matrix Metalloproteinase Regulates Macrophage-dependent Elastolytic Activity and Aneurysm Formation in Vivo. J. Biol. Chem..

[406] Blomkalns A.L., Gavrila D., Thomas M., Neltner B.S., Blanco V.M., Benjamin S.B., McCormick M.L., Stoll L.L., Denning G.M., Collins S.P. (2013). CD14 Directs Adventitial Macrophage Precursor Recruitment: Role in Early Abdominal Aortic Aneurysm Formation. J. Am. Hear. Assoc..

[407] Boytard L., Spear R., Chinetti-Gbaguidi G., Acosta-Martin A.E., Vanhoutte J., Lamblin N., Staels B., Amouyel P., Haulon S., Pinet F. (2013). Role of Proinflammatory CD68 + Mannose Receptor—Macrophages in Peroxiredoxin-1 Expression and in Abdominal Aortic Aneurysms in Humans. Arter. Thromb. Vasc. Biol..

[408] Zagrapan B., Eilenberg W., Prausmueller S., Nawrozi P., Muench K., Hetzer S., Elleder V., Rajic R., Juster F., Martelanz L. (2019). A Novel Diagnostic and Prognostic Score for Abdominal Aortic Aneurysms Based on D-Dimer and a Comprehensive Analysis of Myeloid Cell Parameters. Thromb. Haemost..

[409] Michineau S., Franck G., Wagner-Ballon O., Dai J., Allaire E., Gervais M. (2014). Chemokine (C-X-C Motif) Receptor 4 Blockade by AMD3100 Inhibits Experimental Abdominal Aortic Aneurysm Expansion Through Anti-Inflammatory Effects. Arter. Thromb. Vasc. Biol..

[410] Vandestienne M., Zhang Y., Santos-Zas I., Al-Rifai R., Joffre J., Giraud A., Laurans L., Esposito B., Pinet F., Bruneval P. (2021). TREM-1 orchestrates angiotensin II–induced monocyte trafficking and promotes experimental abdominal aortic aneurysm. J. Clin. Investig..

[411] Iida Y., Xu B., Xuan H., Glover K.J., Tanaka H., Hu X., Fujimura N., Wang W., Schultz J.R., Turner C.R. (2013). Peptide Inhibitor of CXCL4–CCL5 Heterodimer Formation, MKEY, Inhibits Experimental Aortic Aneurysm Initiation and Progression. Arter. Thromb. Vasc. Biol..

[412] Wong K.L., Tai J.J.-Y., Wong W.-C., Han H., Sem X., Yeap W.-H., Kourilsky P., Wong S.-C. (2011). Gene expression profiling reveals the defining features of the classical, intermediate, and nonclassical human monocyte subsets. Blood.

[413] Zawada A.M., Rogacev K.S., Rotter B., Winter P., Marell R.-R., Fliser D., Heine G.H. (2011). SuperSAGE evidence for CD14++CD16+ monocytes as a third monocyte subset. Blood.

[414] Chimen M., Yates C.M., McGettrick H.M., Ward L.S.C., Harrison M.J., Apta B., Dib L.H., Imhof B.A., Harrison P., Nash G.B. (2017). Monocyte Subsets Coregulate Inflammatory Responses by Integrated Signaling through TNF and IL-6 at the Endothelial Cell Interface. J. Immunol..

[415] Auffray C., Fogg D., Garfa M., Elain G., Join-Lambert O., Kayal S., Sarnacki S., Cumano A., Lauvau G., Geissmann F. (2007). Monitoring of Blood Vessels and Tissues by a Population of Monocytes with Patrolling Behavior. Science.

[416] Cros J., Cagnard N., Woollard K., Patey N., Zhang S.-Y., Senechal B., Puel A., Biswas S.K., Moshous D., Picard C. (2010). Human CD14dim Monocytes Patrol and Sense Nucleic Acids and Viruses via TLR7 and TLR8 Receptors. Immunity.

[417] Ghigliotti G., Barisione C., Garibaldi S., Brunelli C., Palmieri D., Spinella G., Pane B., Spallarossa P., Altieri P., Fabbi P. (2013). CD16+Monocyte Subsets Are Increased in Large Abdominal Aortic Aneurysms and Are Differentially Related with Circulating and Cell-Associated Biochemical and Inflammatory Biomarkers. Dis. Markers.

[418] Samadzadeh K.M., Chun K.C., Nguyen A.T., Baker P.M., Bains S., Lee E.S. (2014). Monocyte activity is linked with abdominal aortic aneurysm diameter. J. Surg. Res..

[419] Dale M.A., Ruhlman M.K., Baxter B.T. (2015). Inflammatory Cell Phenotypes in AAAs: Their Role and Potential as Targets for Therapy. Arter. Thromb. Vasc. Biol..

[420] Knappich C., Spin J.M., Eckstein H.-H., Tsao P.S., Maegdefessel L. (2020). Involvement of Myeloid Cells and Noncoding RNA in Abdominal Aortic Aneurysm Disease. Antioxidants Redox Signal..

[421] Dutertre C.-A., Clement M., Morvan M., Schäkel K., Castier Y., Alsac J.-M., Michel J.-B., Nicoletti A. (2014). Deciphering the Stromal and Hematopoietic Cell Network of the Adventitia from Non-Aneurysmal and Aneurysmal Human Aorta. PLoS ONE.

[422] Qin Z., Bagley J., Sukhova G., Baur W.E., Park H.-J., Beasley D., Libby P., Zhang Y., Galper J.B. (2015). Angiotensin II-induced TLR4 mediated abdominal aortic aneurysm in apolipoprotein E knockout mice is dependent on STAT3. J. Mol. Cell. Cardiol..

[423] Hadi T., Boytard L., Silvestro M., Alebrahim D., Jacob S., Feinstein J., Barone K., Spiro W., Hutchison S., Simon R. (2018). Macrophage-derived netrin-1 promotes abdominal aortic aneurysm formation by activating MMP3 in vascular smooth muscle cells. Nat. Commun..

[424] Guo L., Akahori H., Harari E., Smith S.L., Polavarapu R., Karmali V., Otsuka F., Gannon R.L., Braumann R.E., Dickinson M.H. (2018). CD163+ macrophages promote angiogenesis and vascular permeability accompanied by inflammation in atherosclerosis. J. Clin. Investig..

[425] Li J., Xia N., Wen S., Li D., Lu Y., Gu M., Tang T., Jiao J., Lv B., Nie S. (2019). IL (Interleukin)-33 Suppresses Abdominal Aortic Aneurysm by Enhancing Regulatory T-Cell Expansion and Activity. Arter. Thromb. Vasc. Biol..

[426] Zhang Z., Xu J., Liu Y., Wang T., Pei J., Cheng L., Hao D., Zhao X., Chen H.-Z., Liu D.-P. (2018). Mouse macrophage specific knockout of SIRT1 influences macrophage polarization and promotes angiotensin II-induced abdominal aortic aneurysm formation. J. Genet. Genom..

[427] Chen X., Li Y., Xiao J., Zhang H., Yang C., Wei Z., Chen W., Du X., Liu J. (2020). Modulating Neuro-Immune-Induced Macrophage Polarization With Topiramate Attenuates Experimental Abdominal Aortic Aneurysm. Front. Pharmacol..

[428] Malech H.L., DeLeo F.R., Quinn M.T. (2019). The Role of Neutrophils in the Immune System: An Overview. Neutrophil.

[429] Klopf J., Brostjan C., Neumayer C., Eilenberg W. (2021). Neutrophils as Regulators and Biomarkers of Cardiovascular Inflammation in the Context of Abdominal Aortic Aneurysms. Biomedicines.

[430] Klopf J., Brostjan C., Eilenberg W., Neumayer C. (2021). Neutrophil Extracellular Traps and Their Implications in Cardiovascular and Inflammatory Disease. Int. J. Mol. Sci..

[431] Zagrapan B., Eilenberg W., Scheuba A., Klopf J., Brandau A., Story J., Dosch K., Hayden H., Domenig C.M., Fuchs L. (2020). Complement Factor C5a Is Increased in Blood of Patients with Abdominal Aortic Aneurysm and Has Prognostic Potential for Aneurysm Growth. J. Cardiovasc. Transl. Res..

[432] Pagano M.B., Bartoli M., Ennis T.L., Mao D., Simmons P.M., Thompson R.W., Pham C.T.N. (2007). Critical role of dipeptidyl peptidase I in neutrophil recruitment during the development of experimental abdominal aortic aneurysms. Proc. Natl. Acad. Sci. USA.

[433] Langenskiöld M., Smidfelt K., Nordanstig J., Bergström G., Tivesten A. (2020). Leukocyte subsets and abdominal aortic aneurysms detected by screening in men. J. Intern. Med..

[434] Yang P., Li Y., Xie Y., Liu Y. (2019). Different Faces for Different Places: Heterogeneity of Neutrophil Phenotype and Function. J. Immunol. Res..

[435] Memon A., Zarrouk M., Ågren-Witteschus S., Sundquist J., Gottsäter A., Sundquist K. (2019). Identification of novel diagnostic and prognostic biomarkers for abdominal aortic aneurysm. Eur. J. Prev. Cardiol..

[436] Wang Y., Rosen H., Madtes D.K., Shao B., Martin T.R., Heinecke J.W., Fu X. (2007). Myeloperoxidase Inactivates TIMP-1 by Oxidizing Its N-Terminal Cysteine Residue: An Oxidative Mechanism for Regulating Proteolysis during Inflammation. J. Biol. Chem..

[437] Groeneveld M.E., Struik J.A., Musters R.J., Tangelder G.J., Koolwijk P., Niessen H., Hoksbergen A.W., Wisselink W., Yeung K.K. (2019). The Potential Role of Neutrophil Gelatinase-Associated Lipocalin in the Development of Abdominal Aortic Aneurysms. Ann. Vasc. Surg..

[438] Adkison A.M., Raptis S.Z., Kelley D.G., Pham C.T. (2002). Dipeptidyl peptidase I activates neutrophil-derived serine proteases and regulates the development of acute experimental arthritis. J. Clin. Investig..

[439] Abisi S., Burnand K.G., Waltham M., Humphries J., Taylor P.R., Smith A. (2007). Cysteine protease activity in the wall of abdominal aortic aneurysms. J. Vasc. Surg..

[440] Dale M.A., Xiong W., Carson J.S., Suh M.K., Karpisek A.D., Meisinger T.M., Casale G.P., Baxter B.T. (2016). Elastin-Derived Peptides Promote Abdominal Aortic Aneurysm Formation by Modulating M1/M2 Macrophage Polarization. J. Immunol..

[441] Tachieda R., Niinuma H., Ohira A., Endoh S., Hiramori K., Makita S., Nakamura M. (2000). Circulating biochemical marker levels of collagen metabolism are abnormal in patients with abdominal aortic aneurysm. Angiology.

[442] Hance K.A., Tataria M., Ziporin S.J., Lee J.K., Thompson R.W. (2002). Monocyte chemotactic activity in human abdominal aortic aneurysms: Role of elastin degradation peptides and the 67–kD cell surface elastin receptor. J. Vasc. Surg..

[443] Delbosc S., Alsac J.-M., Journé C., Louedec L., Castier Y., Bonnaure-Mallet M., Ruimy R., Rossignol P., Bouchard P., Michel J.-B. (2011). Porphyromonas gingivalis Participates in Pathogenesis of Human Abdominal Aortic Aneurysm by Neutrophil Activation. Proof of Concept in Rats. PLoS ONE.

[444] Meher A.K., Spinosa M., Davis J.P., Pope N., Laubach V.E., Su G., Serbulea V., Leitinger N., Ailawadi G., Upchurch G.R. (2018). Novel Role of IL (Interleukin)-1β in Neutrophil Extracellular Trap Formation and Abdominal Aortic Aneurysms. Arter. Thromb. Vasc. Biol..

[445] Yan H., Zhou H.-F., Akk A., Hu Y., Springer L.E., Ennis T.L., Pham C.T. (2016). Neutrophil Proteases Promote Experimental Abdominal Aortic Aneurysm via Extracellular Trap Release and Plasmacytoid Dendritic Cell Activation. Arter. Thromb. Vasc. Biol..

[446] Eilenberg W., Zagrapan B., Bleichert S., Ibrahim N., Knöbl V., Brandau A., Martelanz L., Grasl M.-T., Hayden H., Nawrozi P. (2021). Histone citrullination as a novel biomarker and target to inhibit progression of abdominal aortic aneurysms. Transl. Res..

